# Structural decoding of netrin-4 reveals a regulatory function towards mature basement membranes

**DOI:** 10.1038/ncomms13515

**Published:** 2016-11-30

**Authors:** Raphael Reuten, Trushar R. Patel, Matthew McDougall, Nicolas Rama, Denise Nikodemus, Benjamin Gibert, Jean-Guy Delcros, Carina Prein, Markus Meier, Stéphanie Metzger, Zhigang Zhou, Jennifer Kaltenberg, Karen K. McKee, Tobias Bald, Thomas Tüting, Paola Zigrino, Valentin Djonov, Wilhelm Bloch, Hauke Clausen-Schaumann, Ernst Poschl, Peter D. Yurchenco, Martin Ehrbar, Patrick Mehlen, Jörg Stetefeld, Manuel Koch

**Affiliations:** 1 Institute for Dental Research and Oral Musculoskeletal Biology, Medical Faculty, University of Cologne, Joseph-Stelzmann-Strasse 52, Cologne 50931, Germany; 2 Center for Biochemistry, Medical Faculty, University of Cologne, Cologne, Joseph-Stelzmann-Strasse 52, Cologne 50931, Germany; 3 Alberta RNA Research and Training Institute, Department of Chemistry and Biochemistry, University of Lethbridge, 4401 University Drive, Lethbridge, Alberta T1K 3M4, Canada; 4 School of Biosciences, University of Birmingham, Edgbaston B15 2TT, UK; 5 Department of Chemistry, University of Manitoba, Winnipeg R3T 2N2, Canada; 6 Apoptosis, Cancer and Development Laboratory, Equipe labellisée ‘La Ligue', LabEx DEVweCAN, Centre de Recherche en Cancérologie de Lyon, INSERM U1052-CNRS UMR5286, Université de Lyon, Centre Léon Bérard, Lyon 69008, France; 7 Center for Applied Tissue Engineering and Regenerative Medicine–CANTER, Munich University of Applied Sciences, Lothstrasse 34, Munich D-80335, Germany; 8 Laboratory of Experimental Surgery and Regenerative Medicine – ExperiMed, Department of Surgery, Clinical Center University of Munich, Nussbaumstrasse 20, Munich D-80336, Germany; 9 Center for Nanoscience—CeNS, Geschwister-Scholl-Platz 1, Munich D-80539, Germany; 10 Laboratory for Cell and Tissue Engineering, Department of Obstetrics, University Hospital Zurich, Schmelzbergstr. 12, Zurich 8091, Switzerland; 11 School of Biological Sciences, University of East Anglia, Norwich Research Park, Norwich NR4 7TJ, UK; 12 Norwich Medical School, University of East Anglia, Norwich Research Park, Norwich NR4 7TJ, UK; 13 Department of Pathology, Robert Wood Johnson Medical School, Piscataway, New Jersey 08854, USA; 14 Department of Dermatology, University Hospital Magdeburg, Magdeburg 39120, Germany; 15 Laboratory of Experimental Dermatology, Department of Dermatology and Allergy, University of Bonn, Bonn 53105, Germany; 16 Department of Dermatology and Venerology, University of Cologne, Cologne 50931, Germany; 17 Institute of Anatomy, University of Bern, Baltzerstrasse 2, Bern 3000, Switzerland; 18 Institute of Cardiovascular Research and Sport Medicine, Department of Molecular and Cellular Sport Medicine, German Sport University Cologne, Cologne 50933, Germany

## Abstract

Netrins, a family of laminin-related molecules, have been proposed to act as guidance cues either during nervous system development or the establishment of the vascular system. This was clearly demonstrated for netrin-1 via its interaction with the receptors DCC and UNC5s. However, mainly based on shared homologies with netrin-1, netrin-4 was also proposed to play a role in neuronal outgrowth and developmental/pathological angiogenesis via interactions with netrin-1 receptors. Here, we present the high-resolution structure of netrin-4, which shows unique features in comparison with netrin-1, and show that it does not bind directly to any of the known netrin-1 receptors. We show that netrin-4 disrupts laminin networks and basement membranes (BMs) through high-affinity binding to the laminin γ1 chain. We hypothesize that this laminin-related function is essential for the previously described effects on axon growth promotion and angiogenesis. Our study unveils netrin-4 as a non-enzymatic extracellular matrix protein actively disrupting pre-existing BMs.

## Abstract (web summary)

Netrins are secreted guidance factors that promote axon outgrowth and orientation during nervous system development. Here the authors present structural and biological evidence that netrin-4 is not a guidance cue per se, but rather functions to modulate laminin-laminin interactions.

## Graphical abstract

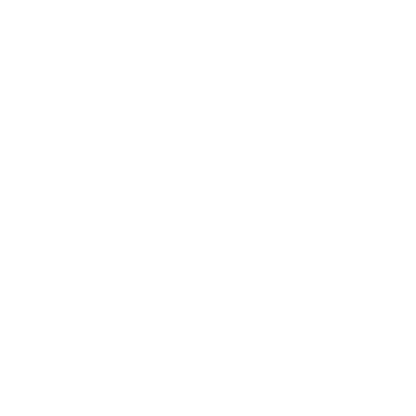

The netrin protein family is comprised of six members, the secreted netrins -1, -3, -4, -5 and the glycosylphosphatidylinositol (GPI)-anchored netrins-G1 and -G2 in mammals and exhibit homology to the N-terminal domains (LN-LEa1-3) of laminin short arms^1^,^2^,^3^,^4^,^5^,^6^,^7^,^8^. Secreted netrins-1,-3 and -5 are homologous to the laminin γ1 chain, whereas netrin-4 (Net4) shares a higher identity to the β1 chain (50%). Netrin-1 (Net1) has been studied in detail and the name netrin, from ‘the one who guides' in Sanskrit was given to this family of laminin-related molecules because of Net1 activity^2^. Net1 one of the first studied secreted guidance factors, plays a major role in promoting both axon outgrowth and axon orientation during nervous system development^9^, as well as in developmental angiogenesis. Given the structural and functional similarities between the axon growth cone and the endothelial tip cell^10^ in both systems netrin-1 function is due to its ability to engage the receptors DCC and UNC5H (that is, UNC5A, UNC5B, UNC5C and UNC5D)^11^,^12^,^13^ and possibly other receptors such as neogenin, A2b, some integrins or DSCAM^14^,^15^,^16^,^17^. Because of the similarity of Net1 and Net4, the majority of studies of Net4 have been focused on describing functions of Net4 based on an assumed interaction of Net4 with DCC or UNC5 and with a guidance function. Works include reported neuronal guidance function^3^ and pro- as well as anti-angiogenic activity^18^,^19^,^20^. The most prominent *in vitro* function of Net4 is the inhibition of endothelial tube-like structure formation when Net4 is applied to endothelial cells seeded on Matrigel^20^. In addition, different cancer cells transfected with Net4 form less-vascularized solid tumours^18^,^19^. In most cases, it was speculated that Net4 acts mainly via binding to Net1 receptors of the DCC/neogenin family and UNC5 family members^19^,^20^,^21^. Recently, the binding epitopes of Net1 for DCC, neogenin and UNC5B were identified^22^,^23^,^24^; however, the proposed binding sites are not conserved within Net4. Of interest, even though these studies have received little attention, it was proposed that Net4 binds to the laminin γ1 chain^25^,^26^. Together with the fact that most *in vitro* effects shown with Net4 were obtained from culture in reconstituted matrix such as Matrigel mainly composed of laminin 111, we hypothesized that the Net4 by interacting with laminin γ1 could impact on the Matrigel *in vitro* and on the basement membrane (BM) *in vivo*.

The BM is a specialized extracellular matrix mainly composed of laminin and collagen IV polymers^27^ as well as a number of additional proteins. This matrix provides a substrate for cell attachment, serves as a barrier, and is a source of growth factors. As an example, in the vasculature the BM is located at the basal side of the endothelium. The vascular BM (vBM) assembles from proteins produced by endothelial as well as perivascular cells (PVCs)^28^. Continuous formation of the vBM along the endothelium stabilizes newly formed vessels and promotes their maturation^28^,^29^. Ablation of the BM core component collagen IV leads to vascular defects, haemorrhages and embryonic lethality at E10.5 in mouse^30^. However, these mice still contain BM-like sheets assembled through laminins^30^. Although, the ultrastructural morphology of BMs is similar between different tissues, variations in protein composition are seen depending on location and during development. Laminins 411 and 511 are the predominant isoforms in the vBM formed by the β1, γ1 and either the α4 or the α5 chain. Laminin 411 is expressed in the vasculature during early developmental stages and deficiency of the laminin α4 chain leads to a vascular phenotype with transient perinatal hemorrhages^31^. In contrast, laminin 511 is found in the vBM of adult mice^32^. Laminin 511, in contrast with laminin 411, contains laminin N-terminal globular (LN) domains on each of its short arms allowing its assembly into a network that is bound to endothelial cell surfaces through integrins, sulfatides and α-dystroglycan. Following laminin assembly at cell surfaces, collagen IV is integrated into the BM^33^,^34^,^35^.

Here, we present the high-resolution structure of Net4 showing unique features that are absent in Net1 as well as in the N-terminal laminin structures and identify the binding epitopes required for a stable Net4/laminin γ1 heterodimeric complex. On the basis of this high-resolution structure, we designed Net4 mutants that negatively impede interaction with laminin γ1, possibly explaining previously observed *ex vivo* effects, on axon outgrowth modulation, and angiogenic activity. Our data also elucidate a mechanism by which Net4 influence the laminin network and play a vital role in angiogenesis. We also propose that the anti-angiogenic property of Net4 *in vivo* is directly related to its impact on BM rearrangement.

## Results

### Net4 has unique structural features absent in other netrins

**Figure 1:**
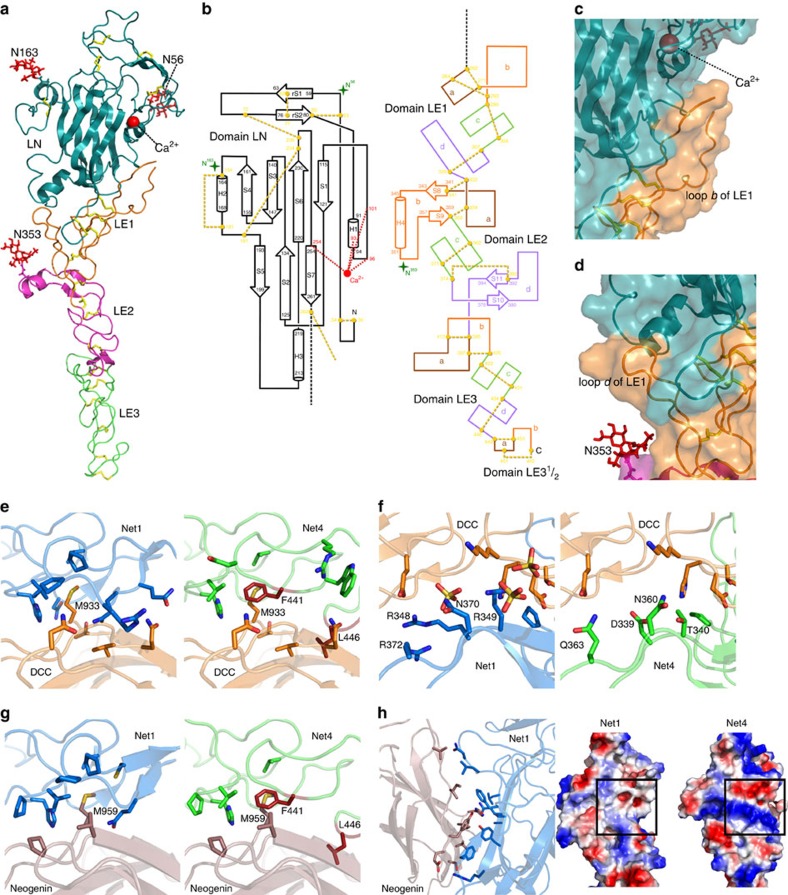
Overall structure of Net4. (**a**) Ribbon model of Net4-ΔC viewed in the ‘open face' orientation. The domains LN (teal), LE1 (orange) LE2 (magenta) and LE3-3^1^/_2_ (green) are coloured accordingly. N-linked glycans are drawn as red sticks, disulfide bridges are drawn as yellow sticks, and the calcium ion as a red sphere. (**b**) Secondary structure diagrams. Disulfide connectivity in domains LN and LE are indicated by yellow dotted lines, visible N-linked glycans by green stars with the Asn residues N^56^, N^163^ and N^353^ highlighted. β-strands are depicted as arrows and the α-helices as cylinders, each labelled with the first and last residue. The individual loop segments are shown in different colour codes. (**c**) Interface between domains LE1 and LN. The KAPG motif is free in solution and not visible in the structure. (**d**) Secondary interface between domains LE1 and LN. The loop *d* segment between Cys307 and Cys329 of subdomain LE1 is in contact with the N-terminus and the loop S5-H3 of domain LN. (**e**) Complex of Net1 (marine; PDB 4OVE) and DCC (orange; PDB 4URT) at site 1 with interacting residues shown at full opacity and the same complex with Net4 (green) replacing Net1. (**f**) Complex of Net1 (marine) and DCC (orange; PDB 4URT) at site 2 with interacting residues shown at full opacity and the same complex with Net4 (green) replacing Net. (**g**) Complex of Net1 (marine) and neogenin (pink; PDB 4PLN) at site 1 with interacting residues shown at full opacity and the same complex with Net4 (green) replacing Net1. Similar to site 1 of the DCC-Net1 complex, F441 (red) of Net4 occludes Met959 of neogenin from the hydrophobic pocket. L456 (red) of Net4 again clashes with the binding partner. (**h**) Net1 (marine) in complex with neogenin (pink) at site 2. Note the difference in the surface charge at the neogenin binding site (indicated by a box) of Net1 (left) and the corresponding site on Net4 (right).

In order to establish the structure-function relationship of Net4, we solved the crystal structure of Net4 lacking the C-terminal domain (Net4-ΔC) at 3.1 Å (Table 1), revealing a head to stalk arrangement of 175 Å in length (Fig. 1a,b). The globular shaped N-terminal domain (LN) forms the head and the three and a half rod-like consecutive LE domains make up the stalk (Fig. 1a,b). The LN domain forms a β-sandwich jelly-roll motif with a front face composed of sheet S5-S2-S7 and a dorsal face composed of sheet S4-S3-S6-S1 (Table 1). The topology of this domain is similar to the N-terminal domain of Net1 (refs 22, 23, 24); however, the closest structural homologue is the short arm of laminin β1 (Supplementary Fig. 1a,b). Significant structural features make this structure unique among netrins. The LN domain is stabilized by a complex pattern of disulfide bridges composed of 12 cysteine residues (Fig. 1a,b). Two disulfide bridges between cysteine residues 72–236 and 191–234, absent in any current netrin or laminin structure, hold the S6-S7 loop in place (Fig. 1b). This loop is adjacent to loop rS1-rS2, which contains the conserved Ser65 (Supplementary Fig. 2a). Interestingly, in laminin β1 (Lmβ1), the replacement of this conserved serine by an arginine allows for the formation of a binary complex between β1 and γ1 laminins; however, the ternary complex formation with the laminin α chain is abolished. Furthermore, there is clear electron density for glycan additions at three asparagine-linked glycosylation sites, Asn56, Asn163, and Asn353 (Supplementary Fig. 2b). The first two glycan chains are located at opposite edges of the LN domain, while Asn353 is located at the loop *b* helix of LE2 and extends towards the bottom of the LN domain (Fig. 1a,b). Remarkably, Net4 is the first member of the netrin family with no N-linked glycan attachment at the dorsal face of the N-terminal domain. Net4-ΔC has a total accessible surface area of ∼28,000 Å^2^ and reveals extensive inter-domain contacts between the LN domain and subdomain LE1 (Fig. 1a,b). Loop *b* of the LE1 domain has a large buried surface area of ∼1,700 Å^2^ with the lateral edge of the LN domain (Fig. 1c). In addition, loop *d* of the LE1 domain is flanked by the Asn353-linked glycan attachment and trapped between the N-terminal segment and the basal helix of the LN domain (Fig. 1d). Together, these extensive contact areas may limit the rotational freedom of the LN domain in comparison with other netrins. Each of the individual LE-subdomains adopts the classical LE-fold^36^ consisting of irregular coil segments and forms a linear extended structure.

### Net4 does not interact with Net1 receptors

As the DCC and UNC5B-binding epitopes of Net1 are absent in the Net4 structure^22^,^23^,^24^, we examined whether Net4 could interact with the ectodomains of DCC, UNC5B, A, C, D and more generally A2b, neogenin as well as DSCAM that have been previously reported to interact with Net1, using solid-phase binding assays and Octet technology. Structural analysis of Net4 and previously published Net1 complex structures reveals incompatibility between the binding residues of Net1 and equivalently positioned residues of Net4 (Fig. 1e–h). For example, Phe441 of Net4 occludes the conserved Met from the hydrophobic pocket in both DCC and neogenin (Fig. 1e,g), and Leu458 of loop *b* in the terminal LE domain of Net4 would preclude interaction with the netrin receptor. The absence of the positively charged residues of Net1 (Arg348, Arg349, Arg351 and Arg372) necessary for a sulfate-mediated interaction with DCC in Net4 would hinder interaction between DCC and Net4 (Fig. 1f). The binding of neogenin to the LN domain involves 10 residues of Net1, which are completely non-conserved in Net4, as presented by the electrostatic surface potential (Fig. 1h). The buried surface area of this interaction is ∼680 Å^2^, and covers a large portion of the LN domain of Net1. None of the residues from Net1 that interact with neogenin are conserved in Net4 (Fig. 1h).

**Figure 2:**
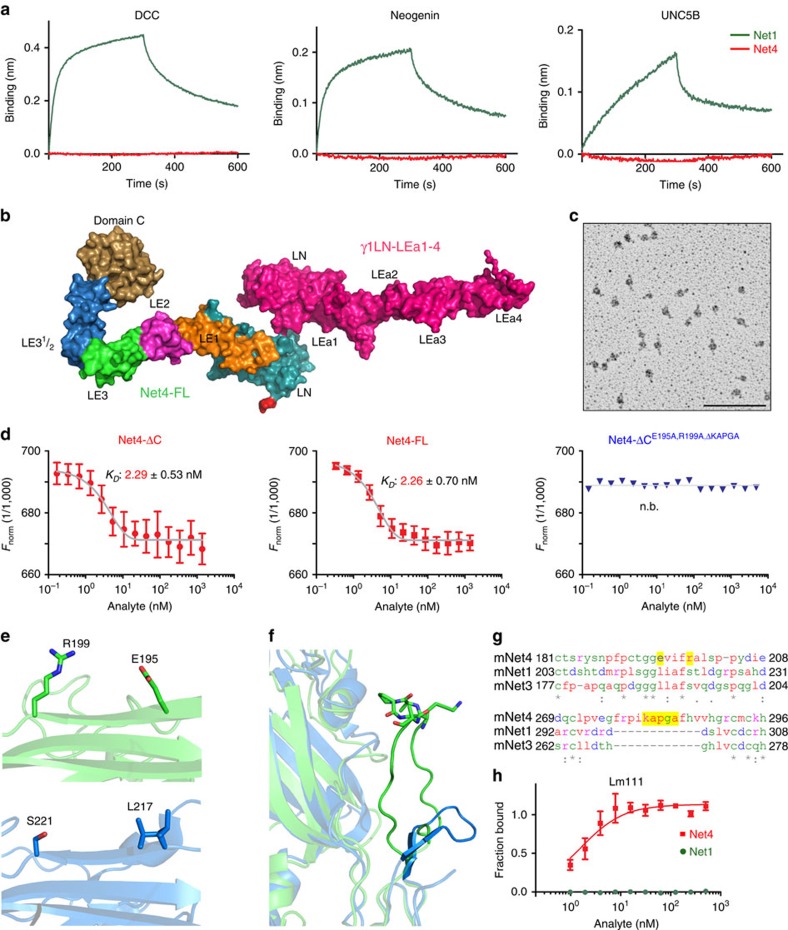
Binding comparison of Net4 and Net1 to netrin receptors and laminin. (**a**) Biolayer interferometry binding kinetic analysis of DCC, neogenin, and UNC5B to Net4 (red) as well as Net1 (green). AHC sensors were coated with the Fc chimera proteins. Association (from 0 to 300 s) and dissociation (from 300 to 600 s) phases are shown for 50 nM of both netrins. (**b**) The SAXS model for the Net4-FL-γ1LN-LEa1-4 complex was generated by firstly calculating models for Net4-ΔC-γ1LN-LEa1-4 complex (Supplementary Fig. 5d) followed by the addition of the LE and globular domain C. This model highlights the role of N-terminal globular domains in the interaction. The agreement between experimentally collected SAXS data and model-derived SAXS data is presented in Supplementary Table 1. (**c**) Rotary-shadowing electron microscopy image of Net4-ΔC-γ1LN-LEa1-4 clearly showing a 1:1 complex mediated via the globular domains. (**d**) Microscale thermophoresis binding analysis of different Net4 mutants to labelled γ1LN-LEa1-4. Binding of Net4 lacking the netrin-specific C domain (Net4-ΔC). Binding of full-length Net4 (Net4-FL). Analysis of the binding of a Net4 mutant (Net4-ΔC^ΔKAPGA^) in which the specific loop *b* (KAPGA) within LE1 was deleted. Binding of a combined Net4 mutant (Net4-ΔC^E195A,R199A,ΔKAPGA^). Error bars, s.d. (*n*=3 independent technical replicates). *K*_D_ values are shown in the graph (n.b., no binding). (**e**,**f**) Laminin-binding epitopes within Net4 (green) compared with the equivalent positions in Net1 (marine). Note loop *b* of LE1 of Net4 has an extensive contact area with the LN domain, and the KAPGA motif (shown in stick notation) is highly flexible. (**g**) Sequence alignment of Net4, Net1 and netrin-3 showing the laminin-binding region. (**h**) Solid-phase binding study of Net4 and Net1 to immobilized laminin γ1 (γ1LN-LEa1-4). Error bars, s.d. (*n*=3 independent technical replicates). Scale bar, (**c**) 100 nm.

While Net1 interacts in the nanomolar range with DCC and UNC5B, Net4 does not show any interaction with any of the proposed Net1 receptors including DCC/neogenin, UNC5 and integrins (Fig. 2a and Supplementary Fig. 3). Sequence alignments of netrin family proteins further highlight that almost all residues involved in Net1 interaction with the canonical netrin receptors are not conserved in Net4 (Supplementary Fig. 4). Together these observations support the view that Net4 does not interact at least directly with the known Net1 receptors, even though we can discard that in some specific settings a protein complex may include Net4 and Net1 receptors.

### Net4 forms a high-affinity complex with laminin γ1

Although full-length Net4 is reported to exist as a dimer^3^ and is therefore believed to bridge two laminin heterotrimers, our crosslinking experiments as well as hydrodynamic studies reveal that full-length Net4 (Net4-FL) forms a high-affinity complex with laminin γ1 (Lmγ1) in a 1:1 molar ratio (Supplementary Fig. 5a–e). The hydrodynamic radius (*r*_H_) from dynamic light scattering (5.77±0.02 nm) and the sedimentation coefficient from analytical ultracentrifugation (AUC, 5.15±0.01 S) confirm unambiguously the existence of an equimolar heterodimeric complex with a molecular mass of 116 kDa. The rotary shadowing experiments reveal a central globular structure with two elongated rods oriented in opposite directions, consistent with a LN domain-mediated major binding interface with both elongated LE subdomains oriented in an antiparallel fashion (Fig. 2c). Small angle X-ray scattering (SAXS) studies of γ1LN-LEa1-4 in complex with full-length Net4 (Net4-FL) yield an extended conformation with a maximum particle size (*D*_max_) of 19 nm (Supplementary Fig. 5d and Supplementary Table 1). Rigid body fitting allows the reconstruction of the assembly of full-length Net4 and laminin γ1 (Net4-FL-γ1LN-LEa1-4) in contact through the LN domains, with the C-terminal domain of Net4 (domain C) curling back over the LE domains. As presented in Fig. 2b, both N-terminal β-sandwiches are oriented like the palms of two hands with the open face sheets S5-S2-S7 (Net4) and S6-S3-S8 (laminin γ1) in contact with each other (Fig. 2b and Supplementary Movie 1). The models were validated extensively by comparing experimentally determined hydrodynamic properties with model derived hydrodynamic properties as presented in the Supplementary Table 1. The comparison of Net4-ΔC and Net4-FL binding to laminin γ1 indicated equal affinities in the range of *K*_D_=2 nM, validating the exclusion of domain C of Net4 in complex formation (Fig. 2d). To decipher individual amino acid residues responsible for Net4-laminin γ1 interactions, we designed mutants based on the three-dimensional structure of Net4 as well as the phylogenetic conservation (Supplementary Fig. 1a,b). A structure-guided deletion of the unique Net4 loop *b* (KAPGA) resulted in a 25-fold reduction of *K*_D_ from 2 nM (Net4-ΔC) to ∼50 nM (Net4-ΔC^ΔKAPGA^) (Supplementary Fig. 6a,b). Thus, Net4-ΔC^ΔKAPGA^ emphasizes the importance of the KAPGA loop in complex formation and gives a rationale for the lower affinity of laminin β1 to laminin γ1 (*K*_D_ of 5 μM)^33^,^35^, as this loop is only present in Net4. Moreover, we identified two residues (E195 and R199) within the LN domain of Net4 involved in laminin γ1 binding. Mutation of these two residues results in a decreased *K*_D_ from 2 to 66 nM (Supplementary Fig. 6c). In addition, mutation of E195 as well as R199 together with deletion of loop *b* (Net4-ΔC^E195A,R199A,ΔKAPGA^) completely abolishes binding in all performed binding assays (Fig. 2d and Supplementary Fig. 6a,b). Crosslinking experiments confirm that full-length Net4 (Net4-FL) exists as a monomer and dimer, because increasing crosslinker concentration results in an increase in dimeric and multimeric Net4-FL (Supplementary Fig. 5a). The *r*_H_ of Net4-FL was determined to be 5.76±0.02 nm which is very similar to that of Net4-FL-γ1LN-LEa1-4 (5.77±0.02 nm) and Net4-ΔC-γ1LN-LEa1-4 (5.40±0.10 nm) complexes but significantly higher than Net4-ΔC (4.60±0.20 nm), implying that full-length Net4 (Net4-FL) behaves as a mixture of monomer and dimer in solution (Supplementary Fig. 5e and Supplementary Table 1). The maximum particle size (*D*_max_) calculated from SAXS data for Net4-FL is significantly higher than the *D*_max_ of Net4-ΔC, but similar to both complexes (Supplementary Fig. 5e and Supplementary Table 1). The AUC studies of Net4-FL provided two peaks corresponding to a monomer and to a dimer for Net4-FL, while Net4-ΔC (ref. 37) and complexes of Net4-FL and Net4-ΔC with γ1LN-LEa1-4 yield only a single peak (Supplementary Fig. 5e). These experiments provide evidence that full-length Net4 (Net4-FL) exists as a mixture of monomer and dimer in solution and that laminin γ1 is capable of destabilizing the Net4-FL dimer. Taken together, our results establish that full-length Net4 binds to laminin γ1 in a 1:1 stoichiometry. In addition, biochemical studies identified different epitopes within Net4 responsible for laminin interaction. Our newly established Net4 laminin-binding mutants can now be used to determine the specific role of the Net4-laminin complex in functional studies because these mutants do not affect cell adhesion properties of Net4 (Supplementary Fig. 6d) indicating their exclusive role in laminin binding. Moreover, comparing the structure of Net4 and Net1 reveals that the laminin-binding epitopes are not conserved within Net1 (Fig. 2e–g) and indeed Net1 does not interact with laminin 111 (Fig. 2h).

### Net4 disrupts pre-existing laminin networks

**Figure 3:**
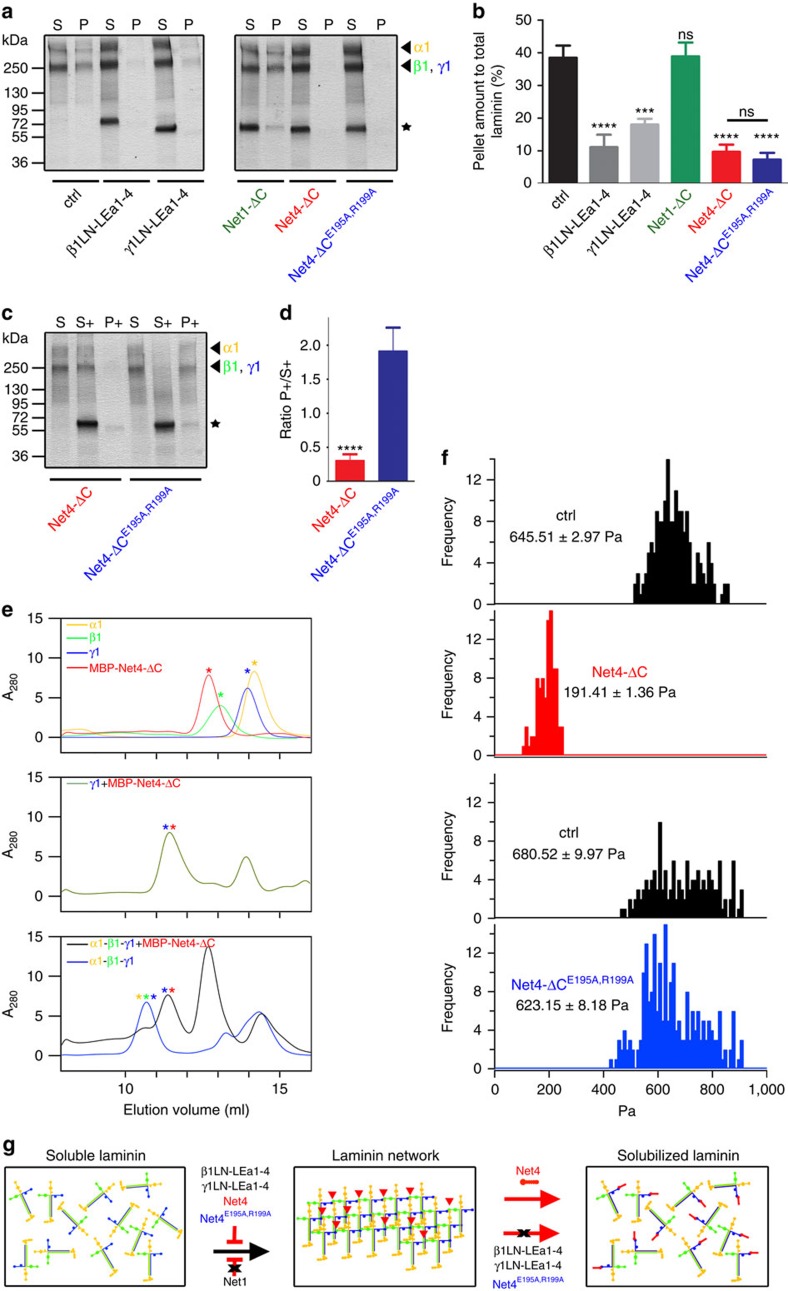
Net4 disrupts pre-existing laminin networks. (**a**) Inhibition of laminin 111 polymerization (Lm111) by human laminin β1 (β1LN-LEa1-4), mouse laminin γ1 (γ1LN-LEa1-4), mouse Net1 (Net1-ΔC), mouse Net4-ΔC and the laminin-binding mutant mouse Net4-ΔC^E195A,R199A^. For the experimental scheme depicting the laminin 111 polymerization assay see Supplementary Fig. 7a. SDS–PAGE analysis of the pellet (P) and the supernatant fraction (S). The position of the laminin chains (arrow heads) and the recombinant proteins (stars) are indicated. (**b**) Densitometric analysis of three independent polymerization experiments and the percentage of the pellet fraction are displayed. (mean±s.d.; *n*=3; ****P<0.00006 (ctrl vs β1LN-LEa1-4, Net4-ΔC and Net4-ΔC^E195A,R199A^) and ***P=0.0004 (ctrl vs γ1LN-LEa1-4); ns, not significant). (**c**) For the method procedure see Supplementary Fig. 7b. The resulting fractions (supernatant of laminin polymerization (S), supernatant after addition of different proteins (S+) and polymer (P+)) were separated by SDS–PAGE, followed by Coomassie Brilliant-Blue staining. Different laminin chains are colour labelled (α1 (yellow), β1 (green) and γ1 (blue)) and added recombinant proteins are indicated with asterisks. (**d**) The ratio between polymer (P+) and supernatant (S+) was determined by densitometric analysis of at least three independent experiments (mean±s.d.; *n*=3; ****P=0.00005). (**e**) N-terminal laminin fragments (LN-LEa1-4) of α1 (yellow), β1 (green) and γ1 (blue) as well as MBP-Net4-ΔC (red) single proteins were analysed using analytical size-exclusion chromatography (top). SEC profile of the laminin fragment γ1LN-LEa1-4 (γ1) in complex with MBP-Net4-ΔC (green, middle). Complexes [α1-β1-γ1 (blue) and α1-β1-γ1+MBP-Net4-ΔC (red)] were analysed by SEC (bottom) revealing that the MBP-Net4-ΔC is able to disrupt the α1-β1-γ1 complex. Asterisks indicate the single proteins within the complex. (**f**) Atomic force microscopy anaylsis of a polymerized Matrigel matrix treated with Net4-ΔC or with the laminin-binding mutant Net4-ΔC^E195A,R199A^. (**g**) Model depicting inhibition of laminin polymerization via laminin β1 (β1LN-LEa1-4), laminin γ1 (γ1LN-LEa1-4), mouse Net4-ΔC, and the laminin-binding mutant mouse Net4-ΔC^E195A,R199A^ but not via mouse Net1 (Net1-ΔC). The pre-existing laminin network can only be solubilized through Net4. Error bars, s.d. (*n*=3 independent technical replicates).

Net4 has been proposed to block laminin polymerization^25^. However, due to its nanomolar binding to the laminin γ1 chain, we hypothesized that Net4 not only blocks laminin polymerization but also disrupts pre-existing laminin network. First, we analysed whether Net4 and the identified laminin-binding mutant (E195A,R199A), which still binds in a nanomolar range to the laminin γ1 chain, are able to block laminin polymerization (Fig. 3a and Supplementary Fig. 7a). Interestingly, Net4-ΔC as well as the laminin-binding mutant Net4-ΔC^E195A,R199A^ blocked laminin polymerization to the same extent as the N-terminal fragments of laminin β1 and γ1 (Fig. 3b). To address our hypothesis that Net4 is able to disrupt pre-existing laminin networks, we performed a comprehensive series of different techniques, including (i) laminin network disruption (Supplementary Fig. 7b), (ii) size-exclusion chromatography and (iii) atomic force microscopy of a polymerized Matrigel matrix. Net4 is able to disrupt pre-existing laminin 111 networks and the ternary node complex formed by the laminin α1, β1 and γ1 chain (Fig. 3c–e). Moreover, Net4 treatment dramatically weakens the stiffness of the Matrigel matrix (Fig. 3f). Thus, all approaches clearly highlight that Net4 is able to disrupt pre-existing laminin networks (Fig. 3c–f). However, the laminin-binding mutant Net4-ΔC^E195A,R199A^ is only able to block laminin polymerization but not to disrupt pre-existing laminin networks (Fig. 3c,d,f). Our results clearly demonstrate that N-terminal laminin fragments and Net4, as proposed previously^25^,^33^ as well as the laminin-binding mutant, are able to block laminin polymerization due to their binding capacity to the N-terminal laminin domain (LN) (Fig. 3g). These results establish that Net4 is able to disrupt pre-existing laminin networks in a non-enzymatic manner, whereas its respective laminin-binding mutant cannot (Fig. 3g).

### Net4 interferes with BM assembly on the cell surface

**Figure 4:**
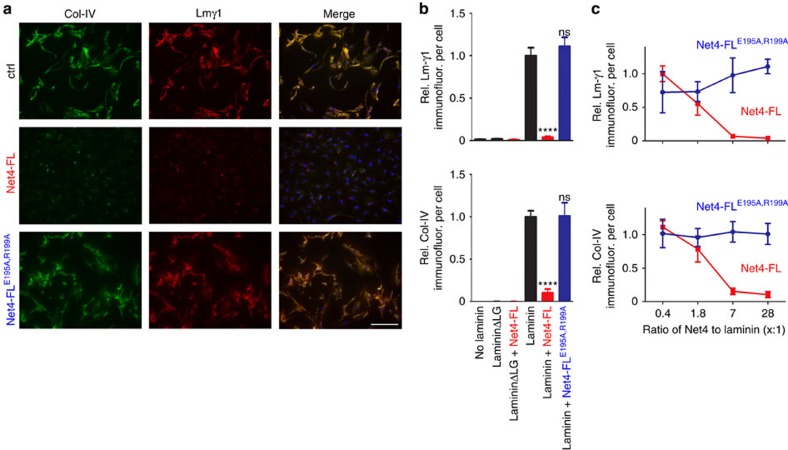
Net4 blocks laminin assembly on the cell surface. (**a**) Within untreated (ctrl), Net4-FL-, and Net4-FL^E195A,R199A^-treated (28-fold excess in molarity compared with laminin) Schwann cell extracellular matrix assembly assay BM components collagen IV (Col-IV, green) and laminin 111 (Lmγ1, red) were stained. Cells were counter-stained for DAPI (blue). (**b**) Staining intensities are displayed relative to the DAPI signal [relative Lmγ1 intensity per cell (left) and relative Col-IV intensity per cell, (right)] (mean±s.d.; *n*=5; ****P<0.00001; ns, not significant). Error bars, s.d. (*n*=5 independent cell cultures). (**c**) Concentration dependent effect of Net4-FL and Net4-FL^E195A,R199A^ on BM formation indicated through measurement of the relative Lmγ1 intensity/cell (left) and relative Col-IV intensity/cell (right). Net4 proteins were added together with the BM components laminin 111, collagen IV and nidogen-1 with increasing molar ratios to laminin 111 (0.4, 1.8, 7, 28-fold excess). Scale bars, (**a**) 200 μm. *P* values were calculated by one-way ANOVA followed by a pairwise comparison using the Holm-Sidak method. ANOVA, analysis of variance.

As Net4 has a high-affinity interaction with laminin γ1, a key component of the BM, we investigated whether Net4 affects BM assembly. As an *in vitro* model, we first took advantage that BM components assemble on Schwann cell surfaces only if laminin 111 heterotrimers interact via their LN domains^34^. Therefore, the stable heterodimeric complex of Net4 with laminin γ1 may prevent efficient BM assembly on Schwann cell surfaces similar to LN domain truncated laminin 111. Schwann cells were incubated with laminin 111, collagen IV and nidogen-1, which normally results in a BM assembly on the cell surface^34^. Deposition of BM components was then monitored via immunofluorescence staining for collagen IV (Col-IV) and laminin (Lmγ1) (Fig. 4a). First, laminin 111 binds to cell surface integrins via its C-terminal LG domains and assembles via the LN domains of the α1, β1 and γ1 chain. Both interactions are necessary for laminin deposition^34^. We observed strong laminin as well as collagen IV staining when treating cells with only laminin 111, collagen IV and nidogen-1 (ctrl) (Fig. 4a,b). After addition of wild-type Net4 (Net4-FL) together with BM components, we could neither detect laminin nor collagen IV deposition by immunofluorescence, whereas treatment with Net4-FL^E195A,R199A^ showed strong laminin as well as collagen IV staining as observed in the control situation (Fig. 4a,b). Moreover, we analysed the influence of different molar ratios of laminin 111 to Net4-FL as well as Net4-FL^E195A,R199A^ (1:0.4, 1:1.8, 1:7 and 1:28). Net4 significantly decreased laminin as well as collagen IV levels on the cell surface at ratios 1:7 and 1:28, whereas the laminin-binding mutant (Net4-FL^E195A,R199A^) had no influence on laminin and collagen IV staining intensities (Fig. 4c). In summary, these results indicate that Net4 can destabilize the polymerization of laminins containing the laminin γ1 chain followed by disassembly of the BM.

### Net4 may affect axon outgrowth through matrix reorganization

**Figure 5:**
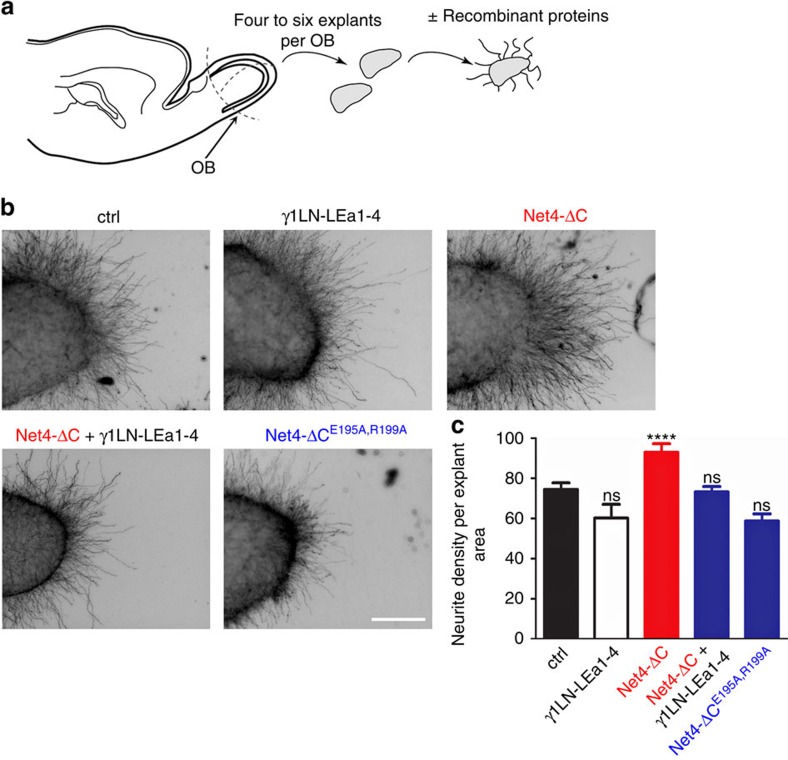
Net4 induces neurite outgrowth in a laminin-dependent manner. (**a**) Diagram depicting the olfactory bulb (OB) explants assay procedure. (**b**) Representative images of untreated (ctrl), laminin γ1 (γ1LN-LEa1-4)-, Net4 (Net4-ΔC)-, Net4 in combination with laminin γ1 (Net4-ΔC+γ1LN-LEa1-4)-, and Net4 laminin-binding mutant (Net4-ΔC^E195A,R199A^)-treated OB explants. (**c**) Statistical analysis of OB explants (mean±s.e.m.; *n*=19; ****P<0.0001, ns, not significant). Error bars, s.e.m. (*n*=19 independent cell cultures). *P* values, two sided *t*-test. Scale bar, 200 μm (**b**).

Net4 has been postulated to play similar roles as Net1, mainly due to their conserved domain features^38^,^39^,^40^. Furthermore, it has been proposed that Net4 acts as an outgrowth promoting cue in *ex vivo* models such as the olfactory bulb neurite outgrowth^3^. We revisited this axon outgrowth function in the light of Net4's impact on the extracellular matrix. Olfactory bulb explants from E15 embryos were cultured in collagen I in the presence of the N-terminal laminin γ1 fragment, Net4-ΔC, Net4-ΔC together with the N-terminal laminin γ1 fragment and the laminin-binding mutant Net4-ΔC^E195A,R199A^, which does not interact with laminin (Fig. 5a). As shown in Fig. 5b, while wild-type Net4 presence is associated with neurite outgrowth, the combination of Net4 with the N-terminal laminin γ1 fragment as well as the laminin-binding mutant was unable to promote axon outgrowth (Fig. 5b,c). These data support the view that, unlike Net1, the axon outgrowth activity of Net4 is not only due to guidance cue functionality per se. It may additionally be due to Net4 ability to disrupt the BM surrounding the olfactory bulb through interactions with laminin-supporting axons to branch through this physical barrier, even though we cannot exclude other interpretations. To further analyse this indirect function, we next focused on the described role of Net4 on angiogenesis.

### Net4 affects angiogenesis through matrix reorganization

**Figure 6:**
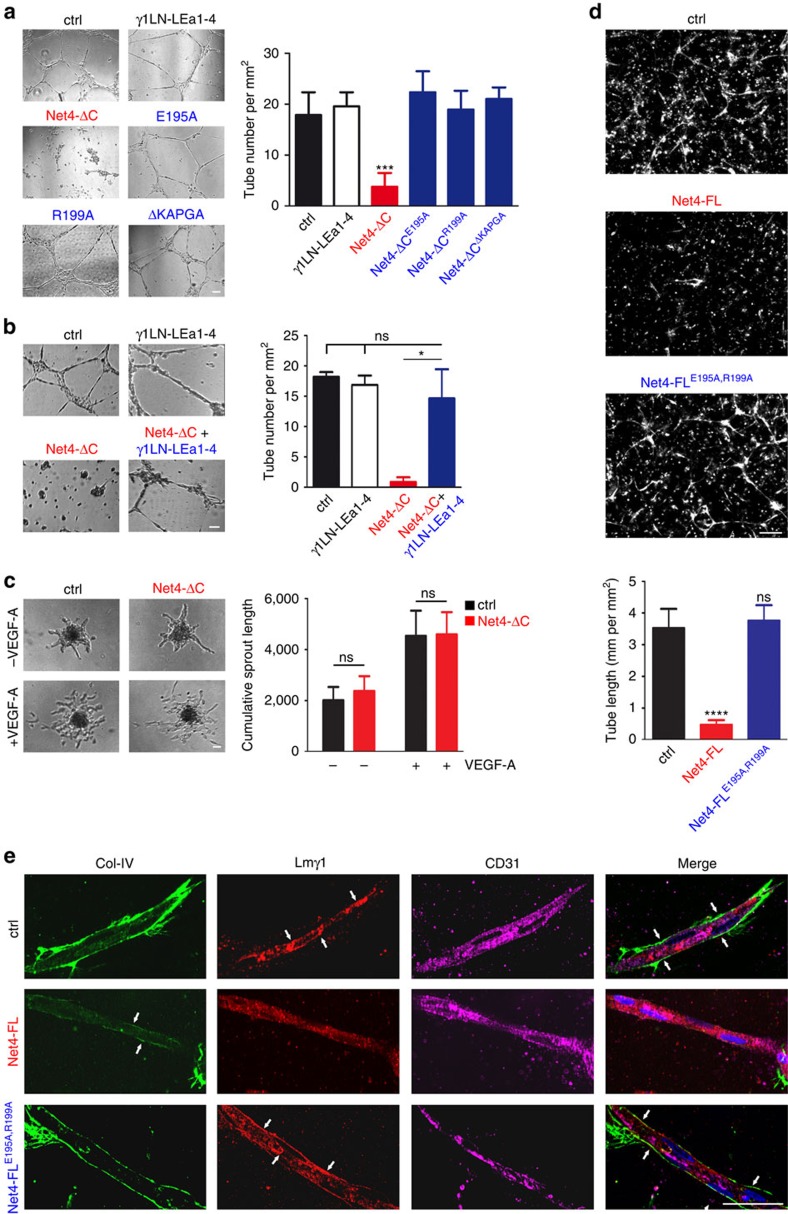
Net4 activity on angiogenesis is dependent on Net4/laminin interaction. (**a**) Analyses of tube-like structure inhibition by Net4-ΔC mutants (Net4-ΔC^E195A^, Net4-ΔC^R199A^ and Net4-ΔC^ΔKAPGA^) impaired in laminin γ1 binding (protein concentrations: 1 μM). Statistical analyses of tube formation via determining the number of cell cluster connections (tube number) (mean±s.d.; *n*=3; ***P=0.0007) (right). Error bars, s.d. (*n*=3 independent cell cultures). (**b**) Blocking of Net4-ΔC inhibited tube formation through equimolar addition of γ1LN-LEa1-4 (1 μM, molar ratio of Net4-ΔC:γ1LN-LEa1-4, 1:1). The laminin fragment γ1LN-LEa1-4 competes with Net4 binding to laminin (mean±s.d.; *n*=3; *P=0.035; ns, not significant). Error bars, s.d. (*n*=3 independent cell cultures). (**c**) Treatment of VEGF-A induced spheroid sprouting of HDMECs embedded in collagen I by Net4-ΔC (1 μM). Analysis of sprouting events (ns, not significant). (**d**) Tube formation analyses of co-cultured endothelial and perivascular-like cells from untreated and Net4-FL- or Net4-FL^E195A,R199A^-treated cultures (30 nM). Representative images of CD31 staining are shown. The tube length (mm per mm^2^) was quantified through the CD31 staining (mean±s.d.; *n*=5; ****P<0.00005). (**e**) Apotome images of Col-IV, Lmγ1 and CD31 stained tubes from control (ctrl), Net4-FL and Net4-FL^E195A,R199A^ treatment are displayed. Error bars, s.d. (*n*=3, (**a**–**c**); *n*=5, (**d**,**e**) independent cell cultures). *P* values, two sided *t*-test. Scale bars, 100 μm (**a**–**d**); 50 μm (**e**).

Most studies investigating the role of Net4 in angiogenesis *in vitro* or *ex vivo* are using artificial matrix for the culture *in vitro*^19^,^20^,^41^,^42^,^43^,^44^. The role of a functional laminin network within artificial matrix such as Matrigel for the formation of tube-like structures has so far not been addressed. A recent study revealed that Net4 inhibits the formation of endothelial tube-like structure at high concentrations (1,000 nM) (ref. 20). To confirm this, Human dermal microvascular endothelial cells (HDMECs) were seeded on Matrigel and different concentrations (0, 200 and 1,000 nM) of Net4 (Net4-ΔC) were added after cell attachment on Matrigel (Supplementary Fig. 8a). Untreated HDMECs formed cell clusters connected by honeycomb-like network structures after 14 h. Upon addition of 200 nM Net4 (Net4-ΔC), endothelial cells were able to form networks, whereas treatment with 1,000 nM Net4 completely abolished tube-like structure formation (Fig. 6a and Supplementary Fig. 8a,b). In addition, HDMEC tube-like structure formation was monitored for 14 h (Supplementary Movies 2 and 3); upon Net4 treatment (1,000 nM) cell morphology was dramatically changed and no network was established. Instead, cells moved together and formed pronounced aggregates within 5 h and remained clustered over the 14 h period (Supplementary Fig. 8c). On the basis of our hypothesis that Net4 disrupts laminin-laminin complexes, we further investigated whether Net4 alters tube-like structure formation in a cell-type independent manner. Upon Net4 treatment, tube-like structure formation in microvascular (HDMEC), macrovascular (HUVEC) and lymphatic (HDLEC) endothelial cell lines was inhibited compared with control (Supplementary Fig. 8d). In addition, Net4 also destabilizes an already established vessel-like network on Matrigel 7 h post treatment followed by increased cell aggregation after 14 h (Supplementary Fig. 8e). In a subsequent step, we further determined whether the exchange of the LN-LEa1 domains within the laminin γ1 chain fragment with the respective laminin-binding domains of Net4 LN-LE1 might lead to gain of function. Both chimeric fragments (chimera 2: Net4LN-LE1-2—γ1LEa3-4 and chimera 3: Net4LN-LE1—γ1LEa2-4) inhibited endothelial tube-like structure formation in the same way as Net4-ΔC, whereas the chimeric fragment (chimera 1: γ1LN-LEa1—Net4LE2-3^1^/_2_) did not show any alteration of endothelial tube-like networks at the concentrations tested (Supplementary Fig. 8f).

To determine whether alterations in the laminin network are the trigger of Net4 mediated inhibition of tube-like structures, we performed the Matrigel assay with the structure-guided Net4 laminin-binding mutants (Net4-ΔC^E195A^, Net4-ΔC^R199A^, and Net4-ΔC^ΔKAPGA^ and Net4-ΔC^E195A,R199A^). HDMECs treated with these laminin γ1 binding mutants established cell clusters with filamentous interconnections in the same manner as untreated and laminin γ1LN-LEa1-4 fragment treated endothelial cells (Fig. 6a, left; Supplementary Fig. 9a,b). Net4 significantly decreased cell cluster interconnections (tube number), whereas tube numbers in γ1LN-LEa1-4- and Net4 laminin-binding mutants-treated endothelial cells were equal to untreated endothelial cells (Fig. 6a, right; Supplementary Fig. 9a,b). The inhibition of tube-like structure formation through Net4 was rescued via pre-incubation of Net4 together with the laminin-binding domain of the laminin γ1 chain (γ1LN-LEa1-4). Analysis of endothelial tube numbers showed no difference between untreated, treatment with γ1LN-LEa1-4, and combined treatment of γ1LN-LEa1-4 together with Net4 (Fig. 6b and Supplementary Fig. 9a,b). This finding demonstrates that the laminin γ1 fragment competes with the interaction of Net4 to the laminin γ1 chain of the laminin 111 heterotrimer. To confirm the cell-independent activity of Net4, we also investigated the influence of Net4 on the tube-like structures^45^ made by the mouse melanoma B16-F1 cell line. Here, Net4 could also block the formation of tube-like structures in a laminin-binding-dependent manner (Supplementary Fig. 10a,b). Furthermore, we analyzed the effect of netrin-4 on endothelial cells in a laminin-free matrix is in agreement with the presented model: addition of netrin-4 had no effect on endothelial cell sprouting in a collagen I matrix (Fig. 6c). To exclude that the observed activity of Net4 is only mediated when Net4 is deposited within the laminin network of the Matrigel matrix, we designed a Net4 protein fused to the N-terminal agrin domain (Agrin-Net4-ΔC). First, we tested the binding activity of Agrin-Net4-ΔC to the laminin γ1LN-LEa1-4 fragment. This construct binds with the same affinity to the laminin γ1LN-LEa1-4 fragment as wild-type Net4 (Supplementary Fig. 6c). Moreover, we tested the binding activity of the N-terminal agrin domain to laminin 111. Here, we took advantage of a previously generated laminin 111 construct lacking the N-terminal domains (LN-LEa1-4) of the laminin γ1 chain (Lm111Δγ1LN-LEa1-4)^34^. As expected, Net4 does not bind to Lm111Δγ1LN-LEa1-4 (Supplementary Fig. 11a), whereas Agrin-Net4-ΔC and the laminin-binding mutant fused to the N-terminal agrin domain (Agrin-Net4-ΔC^E195A,R199A^) interact in the nanomolar range with Lm111Δγ1LN-LEa1-4 (Supplementary Fig. 11b). Treatment of HDMECs with Agrin-Net4-ΔC abolished tube-like structures to the same extend as wild-type Net4 (Net4-ΔC and Net4-FL, Supplementary Figs 9a and 11c). However, the laminin-binding mutant fused to the N-terminal agrin domain as well as the combination of Agrin-Net4-ΔC together with the laminin γ1LN-LEa1-4 fragment significantly blocked the activity of Net4 (Supplementary Fig. 11c). Thus, our data support the view that a formed laminin network within the Matrigel matrix is essential to generate tube-like structures and that the reported inhibitory activity of Net4 is mediated through binding to the LN domain of the laminin γ1 chain and disruption of the BM.

To unravel the physiological significance of our findings we performed a more physiological relevant angiogenesis assay, where endothelial cells were co-cultured with pericytes to form tubes within a laminin-free collagen I matrix^28^. Here, endothelial cells and pericytes form a continuous BM formed by laminin 511 and collagen IV. Treatment of the co-culture with Net4 completely abolished tube formation, whereas the laminin-binding mutant showed no effect (Fig. 6d). Staining of endothelial tubes highlight that laminin as well as collagen IV is not present along the tubes in the Net4 treated set up (Fig. 6e). These results not only suggest that Net4 disrupts the laminin network resulting in the decline of endothelial tubes but also emphasize that the laminin network is the key component of the vBM essential for vessel formation.

### The Net4/laminin interaction destabilizes capillaries *in vivo*

**Figure 7:**
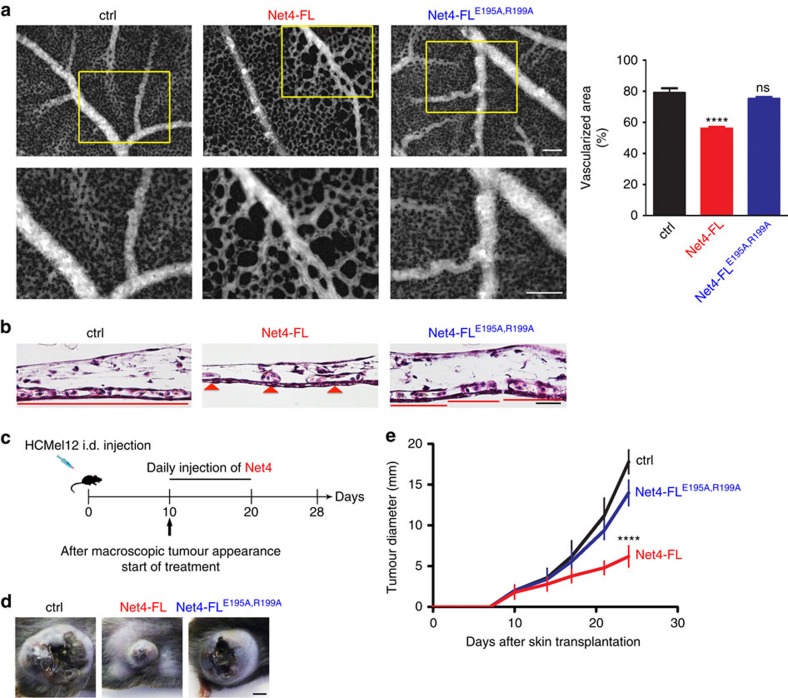
Laminin polymers are essential to maintain capillary networks and tumour progression. (**a**) Visualization of capillaries via FITC-dextran injection of untreated (ctrl), Net4-FL- and Net4-FL^E195A,R199A^-treated CAMs after 48 h. The vascularized area from different protein treatments (left) was determined (see Methods section) at 48 h (mean±s.d.; *n*=5; ****P<0.0001). Error bars, s.d. (*n*=5 independent cell cultures). (**b**) H&E staining of untreated (ctrl) and Net4-FL-, and Net4-FL^E195A,R199A^-treated CAMs after 48 h. Red lines and red arrow heads indicate the capillary density (capillaries are labelled with C). (**c**) Diagram showing the tumour treatment regimen. (**d**) Macroscopic images of melanoma treated daily with 1 μM of control protein (mouse serum albumin, left), Net4-FL (middle), Net4-FL^E195A,R199A^ (right). (**e**) Progression of melanoma treated with Net4-FL and the laminin γ1 binding mutant Net4-FL^E195A,R199A^ (mean±s.d.; *n*=5; ****P<0.0001). Error bars, s.d. (*n*=5 animals, female C57BL/6). *P* values, two sided *t*-test. Scale bars, 100 μm (**a**); 20 μm (**b**); and 5 mm (**d**).

To determine how Net4 affects capillary networks an *ex ovo* chicken Chick Chorioallantoic Membrane (CAM) angiogenesis assay^46^ was performed. Established capillary networks were treated with full-length Net4 (Net4-FL) and the laminin-binding mutant (Net4-FL^E195A,R199A^); blood and micro-capillaries were visualized using FITC-dextran injection 48 h post treatment. In the untreated (ctrl) CAMs, a micro-vascular network appeared 48 h post treatment. Upon addition of Net4, the capillary network dramatically changed as the observed vascularized area was significantly decreased (Fig. 7a). Treating the CAMs with the laminin-binding mutant Net4-FL^E195A,R199A^ had no significant effect. Histological staining (haematoxylin, eosin; H&E) of CAMs to detect the capillary density of untreated and Net4-FL^E195A,R199A^-treated CAMs displayed a dense capillary lining, whereas Net4-treated CAMs revealed fewer and more rounded capillaries (Fig. 7b).

One of the key pathophysiological roles of Net4 associated with its angiogenic activity is its inhibiting effect on tumour progression. Subcutaneously injected cancer cells overexpressing Net4 in mice establish tumours with fewer vessels than tumours established by untransfected cells^18^,^19^. Therefore, we investigated whether the tumour-suppressive activity of Net4 is mediated through binding to laminin. HCMel12 mouse melanoma cells were injected in mice and, upon macroscopic detection; tumour growth was monitored and daily injection of either a control protein (mouse serum albumin), Net4 or its laminin-binding mutant was administered (Fig. 7c). The size of tumours treated with Net4 was reduced by 50% of the size in comparison to the control-treated animals. Moreover, our data establish that the interaction of Net4 with laminin is essential for its tumour-suppressive activity as the Net4 mutant (Net4-FL^E195A,R199A^), affected in laminin binding, had no impact on tumour growth (Fig. 7d,e).

**Figure 8:**
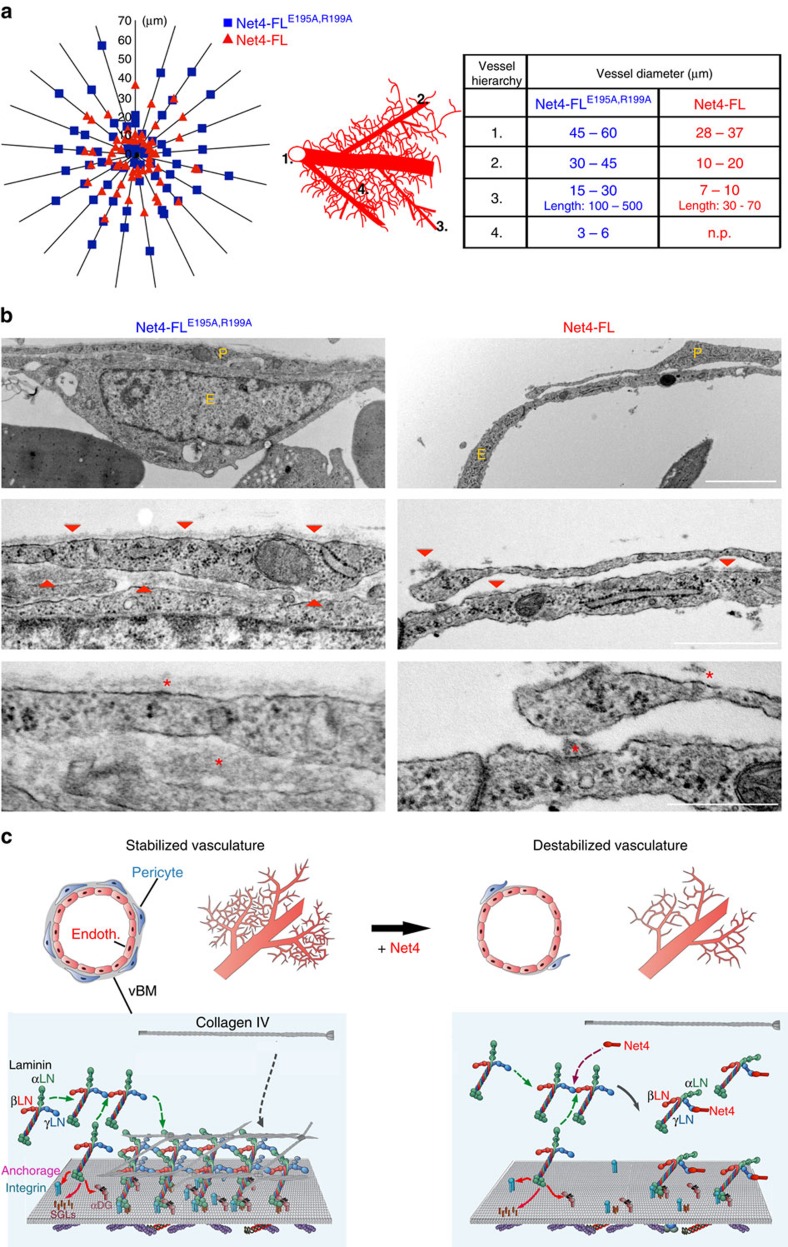
Impact of Net4 on disruption of the laminin network and its effect on vascularization. (**a**) Diagram indicates vessel diameter distribution (left) within Net4-FL^E195A,R199A^-treated CAMs (blue squares), and the Net4-FL group (red triangle). Model showing the common established capillary hierarchy formed within the CAM. Numbers indicate sizes of different vessels in the common established hierarchy (middle). The table displays the diameter of different hierarchical vessels from Net4-FL^E195A,R199A^- (blue) and Net4-FL-treated (red) experiments (right) (n.p., not present). (**b**) Ultrastructural analyses of Net4-FL^E195A,R199A^- (blue) and Net4-FL-treated (red) CAMs using transmission electron microscopy. Images show the capillary endothelium surrounded by perivascular cells. The endothelium E is shown and PVCs are indicated as P. Electron microscopy images highlight the vascular basement membrane (vBM, red arrow heads (middle) and red asterisks (bottom)) between the endothelium and PVCs, which are surrounded by a vBM layer. (**c**) Model of Net4 effect on capillary cell types (endothelium (Endoth.) and pericyte (Pericyte)) and the vBM. Scale bars (**b**), 2 μm (top); 1 μm (middle); 0.5 μm (bottom).

We thus moved back to the *ex ovo* CAM model to further dissect the impact of Net4 on capillaries. We investigated in CAMs treated with Net4 or its laminin-binding mutant, the branching pattern that is highly altered only in the Net4-treated CAM. To highlight the capillary branching hierarchy, the vessel diameters were presented in a radial graph. This analysis clearly indicates that diameters of vessels within Net4-FL^E195A,R199A^-treated CAMs span the full range of diameters found in an established vessel hierarchy. In contrast, vessels in Net4-FL-treated CAMs showed an altered distribution of diameters as large vessels exhibited smaller diameters and small capillaries were not present (Fig. 8a, left). Net4-FL^E195A,R199A^-treated CAMs display a distinct established hierarchy pattern, starting with large vessels (1., 45–60 μm diameter) from which smaller capillaries branch with decreasing diameter (2., 30–45 μm; 3., 15–30 μm; and 4., 3–6 μm). The altered vessel hierarchy in Net4-treated CAMs is clearly demonstrated by decreased diameters as large vessels (1., 28–37 μm) as well as small capillaries (2., 10–20 μm and 3., 7–10 μm) exhibit reduced calibers (Fig. 8a, right). Thus, the laminin–laminin interaction is required to maintain proper capillary networks *in vivo*.

As Net4 appears to disconnect the laminin network *ex ovo*, we hypothesized that the disruption of the laminin network may affect the whole vBM thereby influencing the interaction of pericytes with the endothelium. Therefore, endothelial and perivascular cell distribution was studied in CAMs treated with the laminin-binding mutant (Net4-FL^E195A,R199A^) or Net4. Ultrastructural analyses of Net4-FL^E195A,R199A^-treated CAMs suggested that endothelial cells are closely surrounded by pericytes, whereas Net4 treatment leads to detachment of pericytes from the endothelium with altered pericyte morphology (Fig. 8b, top). Embedding of pericytes into the vBM stabilizes newly-formed vessels. Net4-FL^E195A,R199A^-treated CAMs exhibited a continuous vBM along the endothelium and surrounding pericytes. Upon Net4 treatment, the vBM almost disappeared on endothelial cell surfaces as well as along pericytes (Fig. 8b, middle and bottom). These results indicate that Net4 dissociates laminin heterotrimers from the vBM and disturbs the endothelial/perivascular cell interaction *in vivo*.

## Discussion

Far from its expected role as a guidance cue, a combination of structural, *in vitro* and *in vivo* experiments demonstrate that Net4 behaves in a completely different manner than its well-studied family member Net1. Our data demonstrate that Net4 not only strongly binds to laminin γ1 -thereby blocks efficiently laminin polymerization- but also actively disrupts pre-existing laminin networks in a non-enzymatic manner. Net4 is clearly a unique member among the netrin family, showing non-enzymatic disruptive forces towards matured BMs.

It has been reported that Net4 demonstrates axonal growth activity^3^ and anti-angiogenic activity *in vitro* by inhibiting endothelial tube-like structure formation^18^,^19^,^20^. Furthermore, Net4 overexpression in different cancer cells reveals a decreased vascularization *in vivo*^18^,^19^. Even though we cannot discard that Net4 is having an alternative function via unknown receptor, we propose here that the basis of the effects reported on axonal growth, angiogenesis and tumour progression is due to disruption of pre-existing laminin networks resulting in the disruption of the entire BM. Endothelial assemblies need to form a continuous vBM together with pericytes^28^. Our treatment studies with Net4 confirm this fact due to the fact that the disruption of the vBM results in the decline of endothelial tubes. In terms of the effect of Net4 on neurite outgrowth in the olfactory bulb assay the role of the BM is different. Here, axons need to branch through the BM as a physical barrier and the disruption of this barrier through Net4 supports axonal outgrowth. Interestingly, most of the cellular effects reported on Net4 have been performed using an artificial matrix such as Matrigel that contains mainly laminin 111 (refs 19, 20, 47, 48). As the LN domain of the γ1 chain is the major binding partner of Net4, we hypothesized that it affects the laminin network within the Matrigel matrix instead of directly affecting cell-signalling pathways. Similarly inhibition of blood vessel formation by Net4 is believed to be triggered through binding to the Net1 receptors DCC (DCC, neogenin) and UNC5 (UNC5A-D)^18^,^19^. However, we could not detect direct interactions of Net4 with any of these receptors. Comparison of the high-resolution structures of Net1 (refs 22, 23, 24) and Net4 explains in detail this absence of interaction (Fig. 1e–h and Supplementary Fig. 1a).

Structure based mutations of Net4 in combination with *in vivo* and *ex vivo* assays allowed assignment of Net4 functionality to its laminin-binding capability rather than Net4's interaction with Net1 receptors. Thus, our hypothesis is that Net4 is not a classic ligand activating a membrane receptor with a downstream signalling but rather a regulator of basal membrane maintenance, which indirectly impacts key functions such as axonal growth and angiogenesis. Our study rather support the view that Net4 is a unique protein able to disrupt laminin γ1 containing BMs and this can turn as an useful tool be used to investigate the pivotal role of laminin–laminin interactions in biological processes, for example, muscle formation, somitogenesis and neural tube formation.

In the vasculature, laminin disassembly destabilizes three-dimensional endothelial tubes in an endothelial/pericyte-like cell co-culture via interfering with the formation of a continuous BM along endothelial cell surfaces. Vessel maturation is affected by establishing a continuous BM along endothelial tube-linings. Cooperation between endothelial (ECs) and PVCs induces the production of pivotal matrix components such as nidogen-1, linking the collagen IV structure to the laminin network, and the α5-chain shaping laminin 511, which is the predominant laminin heterotrimer in mature capillaries^28^. The finding that treatment of an EC/PVC co-culture and chicken CAMs with Net4 results in decreased vascularized areas further highlights the essential role of vascular laminin networks in angiogenic processes. Furthermore, the Net4 laminin-binding mutant showed no effect on angiogenesis. Remarkably, Net4 disrupts the entire vBM lying between the endothelium and pericytes as well as the BM surrounding pericytes inhibiting stabilization of the capillary network and formation of the common vessel hierarchy *ex ovo*. Mural cells embedded in the vBM are defined as pericytes^29^. Upon their loss, as demonstrated by ultrastructure analyses of Net4-treated CAMs, the phenotype of pericytes is altered to a more mural cell character. These findings are of potential importance in modulation of cancer progression where blood vessels are not always well organized, but appear destabilized and are often leaky, which hampers drug delivery and increases tumour cell spread^49^,^50^,^51^. The complex between laminin γ1 and Net4 clearly hinders the formation of, or even disrupts, the laminin network. Interestingly, disruption of the vBM leads to detachment of pericytes from the endothelium resulting in the collapse of the entire capillary network (model pictured in Fig. 8c), which may also help to reduce tumour progression. A recent study showed increased permeability in lymphatic vessels upon Net4 overexpression^21^, which fits with the proposed model. Of interest, this model supports the view that the impact of Net4 depends highly on its ability to ‘titrate' laminin and thus an important aspect that remains to be investigated further in animal models is how physiological levels of Net4 actually modulates basal membranes and how in pathological conditions, up- or downregulation of Net4 directly affects the BM and thus the cell behaviour in this Net4 high/low environment. Several studies have shown that Net4 expression is modulated in cancer and as an example is inversely associated with cancer progression^52^ suggesting that Net4 could represent an important modulator of tumoral matrix and thus a putative therapeutic target. Finally, our study not only highlight the key role of laminin networks within the vBM but also shows that Net4 disrupts pre-existing laminin networks and BMs in a non-enzymatic manner.

## Methods

### Recombinant protein expression and purification

Net4-FL, γ1LN-LEa1-4 and serum albumin from *Mus musculus,* (Net4: NP_067295, aa: 20-628, γ1LN-LEa1-4: NP_034813, aa: 34-492, serum albumin: NP_033784, aa: 1-608) and α1LN-LEa1-4, β1LN-LEa1-4 from *Homo sapiens* (α1LN-LEa1-4: NP_005550, aa: 18-453, β1LN-LEa1-4: NP_002282, aa: 31-509) were cloned into a modified pCEP vector with an N-terminal double *Strep* II-tag. Net4-ΔC from *Mus musculus* (NP_067295, aa: 20-462) was cloned into a modified pCEP vector with an N-terminal octa-histidine (His_8_) tag or with an N-terminal double *Strep* II-tag. HEK293 cells were stably transfected followed by screening for subclones with a high level of protein expression. Net4-ΔC, Net4-FL, γ1LN-LEa1-4, α1LN-LEa1-4, and β1LN-LEa1-4 were purified by streptactin-sepharose (IBA) and Net4-ΔC by metal-affinity chromatography using nickel TALON beads (Clontech) followed by the removal of the double *Strep* II-tag and His_8_-tag by thrombin digestion. The purified proteins were then dialyzed against 1 x PBS. Affinity purified proteins were further processed by size-exclusion chromatography (SEC) in TBS buffer (50 mM Tris-HCl, pH 7.5; 200 mM NaCl) on a Superdex 200 column using the ÄKTA FPLC system (GE Healthcare Life Sciences USA) equipped with UV absorbance detector and UNICORN v5.11 software as previously described. The complex of Net4-FL-γ1LN-LEa1-4 and Net4-ΔC-γ1LN-LEa1-4 was purified using SEC by mixing the peak fractions for individual species. Protein concentrations were measured using extinction coefficient and molecular weight values derived from the ProtParam utility^53^ available on the ExPaSy server^54^.

### Design of chimeric constructs and site-directed mutagenesis

To design Net4 containing LNγ1 binding domains we generated chimeric molecules by overlap PCR using the Q5 (New England Biolabs) polymerase. The domains from Net4 (Net4: NP_067295) were replaced by the corresponding laminin γ1 sequences (laminin γ1: NP_034813). The following constructs were amplified, cloned (with an N-terminal double *Strep* 2-tag), verified by sequencing, expressed and purified: Net4LN-LE1-2—γ1LEa3-4 (Net4 aa: 20-394 fused to laminin γ1 aa: 396-492), Net4LN-LE1—γ1LEa2-4 (Net4 aa: 20-331 fused to laminin γ1 aa: 340-492), γ1LN-LEa1—Net4LE2-3^1^/_2_ (Laminin γ1 aa: 44-339 fused to Net4 aa: 332-462) and Net4-ΔC—N-term agrin (NP_067295, aa: 20-462, E195A, R199A, fused to NP_067617, aa: 33–164). Furthermore, the following mutated Net4 molecules were generated: Net4-FL: (NP_067295, aa: 20-628, E195A,R199A), Net4-ΔC^E195A,R199A,ΔKAPGA^: (NP_067295, aa: 20-462, E195A, R199A and Δ281KAPGA285), Net4-ΔC^E195A,R199A^: (NP_067295, aa: 20-462, E195A, R199A and Δ281KAPGA285), and Net4-ΔC^E195A,R199A^—N-term agrin (NP_067295, aa: 20-462, E195A, R199A, fused to NP_067617, aa: 33–164). All produced proteins are shown in Supplementary Fig. 12.

### Structure determination of Net4

Net4-ΔC was applied to a Superdex 200 SEC column (GE Healthcare) equilibrated with 50 mMTris/Tris-HCl, pH 7.5, 0.2 M NaCl at room temperature and the collected peak fractions were immediately concentrated to 1.2 mg ml^−1^ and used for crystallization. Net4-ΔC crystals were grown by hanging-drop vapour diffusion at 293 K by mixing 2 μl of protein solution, 1.6 μl of reservoir solution (20% PEG3350, 0.2 M calcium acetate, and 0.1 M sodium cacodylate pH 6.5), and 0.4 μl of 1 M NDSB-256 from the Hampton HR2-428 additive screen (Hampton Research). Crystals appeared after 1 week and were soaked in mother liquor containing 10% ethylene glycol for 5–10 min before flash freezing in liquid nitrogen. Diffraction data was collected on a Rigaku MM-007HF (*λ*=1.54178 Å) at 100 K. The data set was indexed, integrated and scaled with the HKL2000 suite. The phases were determined by molecular replacement using a polyserine laminin-β homology model (PDB4AQS^55^) from Swissmodel in Phaser^56^. The model was built and refined using Coot^57^, the Phenix Refine and Autobuild programs^58^ and Refmac^59^,^60^.

### Octet-binding studies

Binding of Net4 or Net1 to DCC, neogenin, and UNC5H2 was analysed by biolayer interferometry using the Octet RED96 system (Pall ForteBio). Recombinant human DCC/Fc, mouse neogenin/Fc and rat UNC5H2/Fc chimera were obtained from Bio-Techne. Proteins were stored frozen and diluted into binding buffer (PBS, 0.1% BSA, 0.02% Tween 20). Binding assays were performed in 96-well microtitre plates at 30 °C with orbital sensor agitation at 1,000 r.p.m. Fc chimera proteins (2 μg ml^−1^) were loaded onto anti-human Fc (AHC) biosensors for 10 min. Biosensors were washed with binding buffer for 60 s and placed in wells containing the various netrins (50 nM in binding buffer). Association was observed for 10 min, then biosensors are incubated in binding buffer for additional 10 min to observe dissociation of the complex. Uncoated biosensors were run in parallel for non-specific binding. Binding curves were analysed by Octet User software (ForteBio). Three independent technical replicates were performed.

### Microscale thermophoresis binding assay

Binding of the labelled Laminin γ1 short arm fragment (γ1LN-LEa1-4) to Net4 variants and its respective mutants were measured using microscale thermophoresis. A range of concentrations of the required ligands (from 0.08 to 1,000 nM for Net4 and from 0.45 to 4,000 nM for Net4 mutants) were incubated with 2 nM of γ1LN-LEa1-4 in the assay buffer (50 mM Tris/HCl, pH 7.4, 150 mM NaCl, 2 mM CaCl_2_ supplemented with 3% BSA) for 10 min. Samples were loaded into NanoTemper glass capillaries and microscale thermophoresis was carried out using 10% light-emitting diode power and 80% MST power on a NanoTemper monolith NT.115 (ref. 61). *K*_D_ values were calculated from independent technical triplicate experiments using the mass action equation via the NanoTemper software.

### Laminin network disruption assay

The laminin polymerization-blocking assay was performed as described in the Supplementary Fig. 9b and the laminin polymer disruption assay setup is described in the Supplementary Fig. 9c. Mouse laminin 111 (Sigma-Aldrich, Germany) was diluted to 0.35 μM in 50 mM Tis-HCl, pH 7.4; 150 mM NaCl; 0.1% Triton-X100; 1 mM CaCl_2_ and incubated at 37 °C for 3 h to polymerize. Afterwards, polymerized laminin was centrifuged (10,000*g*; 15 min) to separate formed laminin polymer from non-polymeric laminin. Different proteins (Net4-ΔC, Net4-ΔC^E195A,R199A^) were added at a concentration of 2.1 μM respectively to the laminin polymer at 37 °C for additional 3 h. Samples were centrifuged and all three fractions (supernatant (S), supernatant+ (S+), pellet+ (P+); Supplementary Fig. 7b) were analysed by SDS–PAGE (SDS–polyacrylamide gel electrophoresis) followed by fast Coomassie Blue staining (Thermo Scientific, Germany). Band intensities were determined using the ImageJ software and ratios between the polymer fraction (P+) and supernatant (S+) are displayed.

### Laminin assembly on Schwann cell surface

Rat Schwann cells were cultured in Dulbecco's Eagle medium, 10% fetal calf serum (Atlanta Biological), neuregulin (0.5 μg ml^−1^, Sigma), forskalin (0.2 μg ml^−1^, Sigma) and penicillin-streptomycin. Cells at passage 22-29 were seeded onto 24-well dishes (Sigma) and incubated with collagen IV, nidogen-1 and different laminin constructs. Additionally, cells were treated with 28-fold excess compared with laminin of Net4-FL, Net4-FL^E195A,R199A^ at 37^o^C for 1 h. Schwann cells were rinsed with PBS and fixed in 3% paraformaldehyde for 20 min followed by blocking with 5% goat serum in PBS overnight at 4 °C. Cells were incubated with anti-laminin γ1 (1:200, Millipore, MAB1920) and anti-collagen IV (10 μg ml^−1^, Millipore, AB756P) at room temperature for 1 h. Alexa Fluor 647 goat anti-mouse and Alexa Fluor 488 goat anti-rabbit secondary antibodies (Molecular Probes) were used at 1:100 and counterstained with 4′,6-diamindino-2-phenylindol. Digital images of protein immunofluorescene levels were recorded with IPLAB 3.5 software (Scanalytics) and quantified with IMAGE J. Validation information for commercial antibodies is available on the manufacturers' websites. All experiments were performed in groups of five independent cultures and repeated at least twice.

### Neurite outgrowth assay

Olfactory bulb (OB) explants were isolated from rat embryos E15 (*n*=19 Embryos at E15 were collect from pregnant Sprague Dawley female rat). OB explants were embedded in Collagen I isolated from rat tail and cultured with 28 nM of Net4-FL or Net4-FL^E195A,R199A^ for 24 h. Explants were then fixed in 4% PFA at room temperature for 1 h and permeabilized with PBST (PBS, 0.1% Triton-X100) for 10 min. Next, explants were blocked with PBST, 1% normal donkey serum (Jackson Immunoresearch) at room temperature for 2 h and incubated with mouse polyclonal Tuj1 antibody, 1/500 (Babco, MMS435P) diluted in PBST at 4 °C overnight. After 3 times washing with PBST, explants were incubated with Alexa488 donkey anti mouse (1/500; Life Technologies, A21202) at room temperature for 1 h, followed by 3 washes in PBST and mount with mowiol. Explants were imaged with Axiovert200M, Zeiss. Neurite and explant area were analysed with FIJI in order to determine neurite density (neurite area/explant area). Validation information for commercial antibodies is available on the manufacturers' websites. All experiments were performed in groups of five independent cultures and repeated at least twice.

### Cell culture

HEK293 EBNA cells as well as the mouse melanoma B16-F1 cell line were obtained from ATCC. HDMEC and HDLEC were obtained from Promocell; HUVEC from Lonza. The endothelial cells were cultured in Vasculife VEGF-Mv Medium Complete Kit (Cell Systems) according to the manufacturer´s instructions. The mouse melanoma cell line (HCMel12) was kindly provided by T. Tüting (University of Magdeburg)^62^. The mouse melanoma cell lines HCMel12 and B16-F1 as well as HEK293 EBNA cells were cultured in Dulbecco´s Modified Eagle Medium (Gibco) supplemented with 10% fetal bovine serum. PVCs were isolated from meninigi of the Anxa5-LacZ reporter mouse strain^63^. PVCs were cultured in DMEM supplemented with 10% FCS and used at passages 28–40.

### Tube-like formation assay

Cells were seeded at a density of 25,000 cells per well onto chamber slides (Labtek) or ibidi slides (ibidi) previously coated at 37 °C with Matrigel BM matrix (Corning, New York, USA) for 30 min to allow polymerization. For the tube formation assay, the indicated proteins were added directly after seeding the cells. Cells were incubated for 14 h. For the tube regression assay proteins were added 7 h after seeding the endothelial cells, which had established a tubular network. Recombinant proteins were added at the concentrations indicated and in terms of endothelial cells treatment PDGF as well as VEGF (Biomol) was added at final concentrations of 10 ng ml^−1^. Tube formation was documented by bright-field microscopy using a Nikon Eclipse TE2000-U microscope. In order to investigate the time course of tube formation, we performed time-lapse microscopy using an Olympus IX81. All experiments were performed in groups of five independent cultures and repeated at least three times.

### Three-dimensional culture in collagen I matrix

Endothelial and pericyte-like cells (HUVECs/PVCs) were co-cultured in 2% collagen I gel. Cultures were incubated in ECGM2 medium supplemented with PDGF-BB and VEGF (10 ng ml^−1^ each) for 6 days. Recombinant Net4-FL and Net4-FL^E195A,R199A^ were added to the three-dimensional gel solution at indicated concentration. Medium (supplemented with PDGF-BB, VEGF, and Net4-FL or Net4-FL^E195A,R199A^) was replaced every 48 h. Samples were fixed with Dent's fixative and immunostained for collagen IV (1:500; AB769, Chemicon), laminin γ1 (1:500; kind gift from U. Mayer, University of East Anglia), and human CD31 (1:500; 555444, BD Biosciences). DNA was stained by Hoechst 33258. Overview pictures were taken from each sample by using a Zeiss Axioplan microscope and average tube length was calculated by the Volocity software. Optical section images were taken by a Zeiss Apotome microscope and processed by Axiovision program. All experiments were performed in groups of five independent cultures and repeated at least three times. Validation information for the laminin γ1 antibody is presented in the work of Mayer *et al*.^64^

### Chick chorioallantoic membrane angiogenesis assay

The *ex ovo* culture of the chicken embryos was adapted from Auerbach *et al*.^46^ Briefly, fertilized Ross chicken eggs (Wüthrich Brüterei AG, Switzerland) were incubated at 37 °C for 3 days in humidified atmosphere. On day 3, the eggs were carefully opened and their content was transferred into 100 × 20 mm Petri dishes (Corning, Switzerland). The chicken embryos were incubated in the same conditions for 6 more days. On day 9, the polyethylene glycol (PEG) hydrogels containing no protein and different proteins (Net4-FL, Net4-FL^E195A,R199A^) were placed on the surface of the CAM. 15 μl PEG hydrogels were formed by Factor XIII_a_-catalysed crosslinking of two eight-arm PEG components (8-PEG–Gln and 8-PEG–MMP_sensitive_–Lys) in 50 mM Tris pH 7.6, 50 mM CaCl_2_ buffer to a final PEG concentration of 2% (w/v). Each hydrogel contained 4.25 μg of recombinant Net4-FL, or Net4-FL^E195A,R199A^, which were added to the hydrogel mixture before polymerization. After 48 h of treatment, 100 μl of 2.5% FITC-dextran (MW 20,000, Sigma-Aldrich, Switzerland) in 0.9% NaCl solution were injected intravenously in the CAM. The vasculature was visualized by fluorescence microscopy and images around the treatment site were collected. All experiments were performed in groups of five independent cultures and repeated at least twice.

The images collected by fluorescence microscopy were analysed using the ImageJ software (ImageJ 1.48, http://imagej.nih.gov/) using a script based on the quantification method developed by Blacher *et al*.^65^ Background of each image was evened out and the contrast was enhanced. Then the threshold was adjusted manually do discriminate the vascularized area from the non-vascularized area, and the image was transformed into a binary image. A series of filters were applied then to refine the capillary structures. Region of interests (ROIs) were set manually to exclude second-order and higher-ranking blood vessels and regions with underlying blood vessels. The vascularized areas within the ROIs, as well as the total ROI area, were measured. The obtained values were used to determine the distribution vascularized areas in the capillary network. All experiments were performed in groups of five independent cultures and repeated at least three times.

### Immunohistochemistry of the CAM

After live imaging, the CAMs were fixed with 4% para-formaldehyde solution by applying the solution both on top and beneath the CAM membranes and incubated at room temperature for 1 h. The area around the treatment site was carefully cut out and placed between cellulose sheets before being processed for paraffin embedding. Tissue sections of 4 μm were cut with a microtome and mounted on glass slides for haematoxylin and eosin staining.

### Transmission electron microscopy analyses of CAM capillaries

After live imaging, CAMs were fixed with 2.5% glutaraldehyde (buffered with 0.1 M sodium-cacodylate, pH 7.4, 540 mOsm.) by applying the solution both on top and beneath the CAM membranes. They were post-fixed in 1% OsO_4_ (0.1 M sodium-cacodylate, pH 7.4, 340 mOsm), dehydrated in ethanol and embedded in Epon 812 (Fluka, Buchs, Switzerland). Semi-thin sections were prepared, stained with toluidine blue, and analysed with a light microscope^66^,^67^. Representative areas were selected and further imaged by transmission electron microscopy. Tissue samples from groups of three of three independent biological replicates were analysed.

### Mice model of melanoma

The melanoma HCmel12 cell line, which spontaneously develops lung metastases after intracutaneous injection in immunocompetent C57BL/6 mice, was established from a primary Hgf-Cdk4R24C melanoma^62^. For tumour transplantation 2 × 10^5^ cells in 100 μl 1 × PBS were injected intracutaneously into the flanks of C57BL/6 mice (female, *n*=5, 8 weeks of age) and tumour development was monitored by visual inspection and palpation. When tumours exceed 2 mm groups of five mice were peritumorally injected daily with 1 μM of mouse albumin (ctrl), Net4-ΔC, Net4-FL and Net4-FL^E195A,R199A^ for 10 consecutive days. Tumour sizes were measured every second day and recorded as mean diameter in mm. Mice with tumours exceeding 20 mm diameter were killed. All experiments were performed in groups of five mice and repeated independently at least twice.

### Statistics

Statistical analysis was performed using the GraphPad Prism software (GraphPad Prism 5.00). The two sided *t*-test was used for statistical analysis, statistical analyses of the Schwann cell assay was performed using the one-way analysis of variance followed by a pairwise comparison using the Holm–Sidak method. Results are displayed as mean±s.d. except the one of the neurite outgrowth assay, in which the results are displayed as±s.e.m.

### Study approval

All animal experiments were performed in accordance with the German laws for animal protection and were approved by the State Office North Rhine-Westphalia (LANUV), Germany or by the CECCAP, France.

### Data availability

The data that support the findings of this study are available from the corresponding authors upon request. Coordinates and structure factors for Net-4ΔC have been deposited in Protein Data Bank under accession code 4WNX.

## Additional information

**How to cite this article:** Reuten, R. *et al*. Structural decoding of netrin-4 reveals a regulatory function towards mature basement membranes. *Nat. Commun.* **7,** 13515 doi: 10.1038/ncomms13515 (2016).

**Publisher's note**: Springer Nature remains neutral with regard to jurisdictional claims in published maps and institutional affiliations.

## Supplementary Material

Supplementary Information Supplementary Figures 1-12, Supplementary Table 1, Supplementary Methods and Supplementary References

Supplementary Movie 1 Three-dimensional rotation of the Net4-FL-γ1LN-LEa1-4 SAXS complex. The movie represents the high-resolution model of Net4-FL (multi colour) / γ1LNLEa1-4 (magenta colour) complex.

Supplementary Movie 2 Time-lapse movie showing untreated HDMEC tube-like structure formation on Matrigel over 14 h. HDMEC cells start to form cell clusters followed by the generation of filamentous interconnections between the clusters. The process of endothelial tube-like structure formation is finished after 12 hours. Afterwards, filamentous structures start to decline.

Supplementary Movie 3 Time-lapse movie showing Net4-δC-treated HDMEC tube-like structure formation on Matrigel over 14 h. HDMEC cells seeded on Matrigel and treated with 1 μM Net4 migrate to each other and form aggregates. Here, no filamentous interconnections are generated and the cells remained clustered.

Peer Review

## Figures and Tables

**Table 1:** Data collection and refinement statistics.

	**Net4-ΔC**
*Data collection*	
Space group	C121
Cell dimensions	
*a*, *b*, *c* (Å)	107.755, 74.049, 74.672
α,β,γ (°)	90, 96.072, 90
Resolution (Å)	46.00-3.07 (3.12-3.07)*
No. reflections	39327 (1952)
*R*_merge_	0.194 (0.776)
*I*/σ*I*	6.10 (2.0)
Completeness (%)	99.9 (99.8)
Redundancy	3.5 (3.5)
	
*Refinement*	
*R*_work_/*R*_free_	0.2198/0.2626
No. atoms	3,382
Protein	3,259
Ligand/ion	96
Water	27
*B*-factors	45.90
Protein	44.10
Ligand/ion	110.30
Water	32.60
R.m.s. deviations	
Bond lengths (Å)	0.003
Bond angles (°)	0.77
Ramachandran Statistics	
Favoured (%)	92.2
Disallowed (%)	0.0

^*^Values in parentheses are for highest-resolution shell.
