# Supplementary material for: Structural decoding of netrin-4 reveals a regulatory function towards mature basement membranes
Source: Nat Commun. 2016 Nov 30;7:13515. doi: 10.1038/ncomms13515 (PMC5141367; doi:10.1038/ncomms13515)
Supplement: Supplementary Information — Supplementary Figures 1-12, Supplementary Table 1, Supplementary Methods and Supplementary References [file ncomms13515-s1.pdf]

a

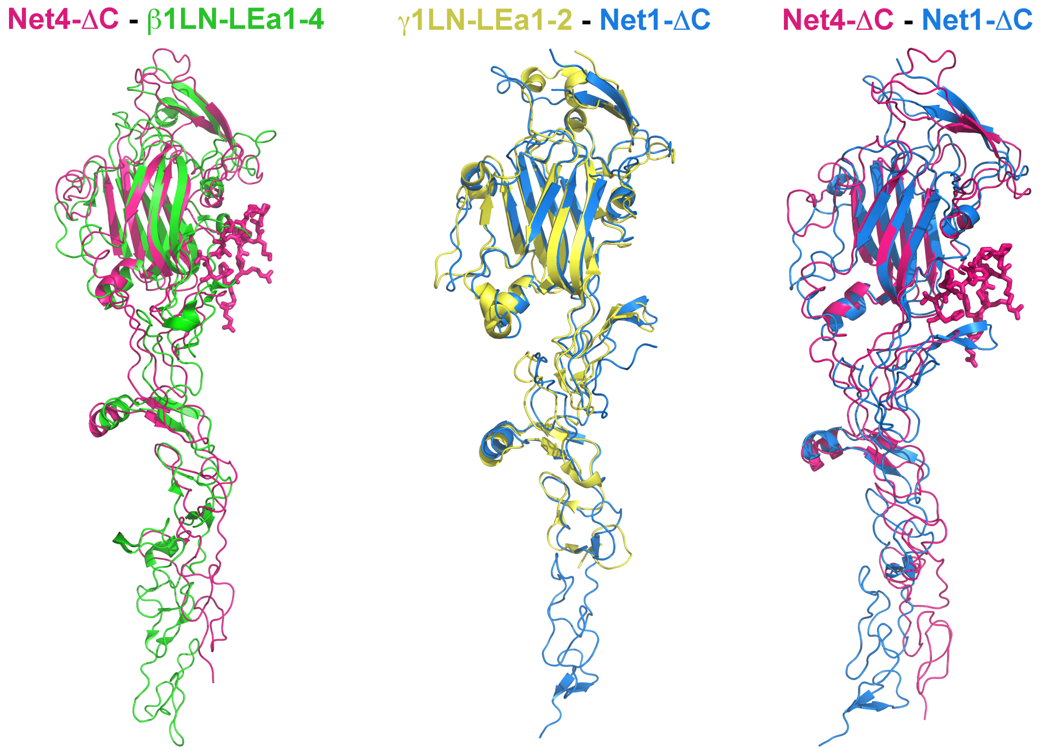

b

```
mNet4    GLNGVAGANSRCEKACNPRMGNLALG..RKLRADTMCGQNATELFCFYSENADLTCRQPK
mLmβ1    ..QEPEFSYGCAEGSCYPATGDLLIGRAQKLSVTSTCGLHKPEPYCIVSHLQEDKK.CFI
mNet1    AQPDPCSDENGHPRRCIPDFVNAAFG..KDVRVSSTCGRP.PARYCVVSER..GEERLRS
mLmγ1    AAMDECADEGGRPQRCMPEFVNAAFN..VTVVATNTCGTP.PEEYCVQTG...VTGVTKS

mNet4    CDKCNAAHSHLAHPPSAMADSSFRFP....RTWWQSA......EDVHREKIQLDLEAEFY
mLmβ1    CDSRDPYHETLNPDSHLIENVVTTFAPNRLKIWWQSE......NGVENVTIQLDLEAEFH
mNet1    CHLCNSSDPKKAHPPAFLTDLNNPHN....LTCWQSE...NYLQFPHNVTLTLSLGKKFE
mLmγ1    CHLCDAGQQHLQHGAAFLTDYNNQAD....TTWWQSQTMLAGVQYPNSINLTLHLGKAFD

mNet4    FTHLIMVFKSPRPAAMVLDRSQDFGKTWKPYKYFATNCSATFG......LEDDVVKKGAI
mLmβ1    FTHLIMTFKTFRPAAMLIERSSDFGKAWGVYRYFAYDCESSFPGIS...TGPMKKVDDII
mNet1    VTYVSLQFCSPRPESMAIYKSMDYGRTWVPFQFYSTQCRKMYNRPHRAPITKQ.NEQEAV
mLmγ1    ITYVRLKFHTSRPESFAIYKRTREDGPWIPYQYYSGSCENTYSKANRGFIRTGGDEQQAL

                       *  *
mNet4    CTSRYSNPFPCTGGEVIFRALSP.PYDIENPYSAKVQEQLKITNLRVRLLKRQSCPCQIN
mLmβ1    CDSRYSDIEPSTEGEVIFRALDP.AFKIEDPYSPRIQNLLKITNLRIKFVKLHTLGDNLL
mNet1    CTDSHTDMRPLSGGLIAFSTLDGRPSAHDFDNSPVLQDWVTATDIRVAFSRLHTFGDENE
mLmγ1    CTDEFSDISPLTGGNVAFSTLEGRPSAYNFDNSPVLQEWVTATDIRVTLNRLNTFGDEVF

mNet4    DLNAKPHHFMHYAVYDFIVKGSCFCNGHADQCLPVEGFRPIKAPGAFHVVHGRCMCKHNT
mLmβ1    DSRMEIREKYYYAVYDMVVRGNCFCYGHASECAPVDGVNEEVEG....MVHGHCMCRHNT
mNet1    DDSELARDSYYYAVSDLQVGGRCKCNGHAARCVRDR............DDSLVCDCRHNT
mLmγ1    NDPKVLKS.YYYAISDFAVGGRCKCNGHASECVKNE............FDKLMCNCKHNT

mNet4    AGSHCQHCAPLYNDRPWEAADGRTGAPNECRT
mLmβ1    KGLNCELCMDFYHDLPWRPAEGRN..SNACKK
mNet1    AGPECDRCKPFHYDRPWQRATARE..ANECVA
mLmγ1    YGVDCEKCLPFFNDRPWRRATAES..ASECLP
```

Supplementary Figure 1. Alignment of netrins and laminins

(**a**) 3D structure comparison of Net4-ΔC-β1LN-LEa1-4, γ1LN-LEa1-2-Net1-ΔC, and Net4-ΔC-Net1-ΔC.

(**b**) The LN and LE1 domain without the signal peptide of mouse Net4 (mNet4, NP_067295), mouse laminin β1 (mLmβ1, NP_032508), mouse Net1 (mNet1, NP_032770), and mouse laminin γ1 (Lmγ1, NP_034813) were aligned using T-coffee[24] and formatted with ESPRIPT (espript.ibcp.fr/ESPript/cgi-bin/ESPript.cgi). Asterisks indicate the amino acids involved in binding between Net-4 and Lmγ1. Interestingly, the Net4 residues E195 and R199 are also conserved in the Lmβ1 chain, however the KAPGA loop in the LE1 domain is unique to the Net4 protein. Asterisks indicate the conserved amino acids mutated in our study.

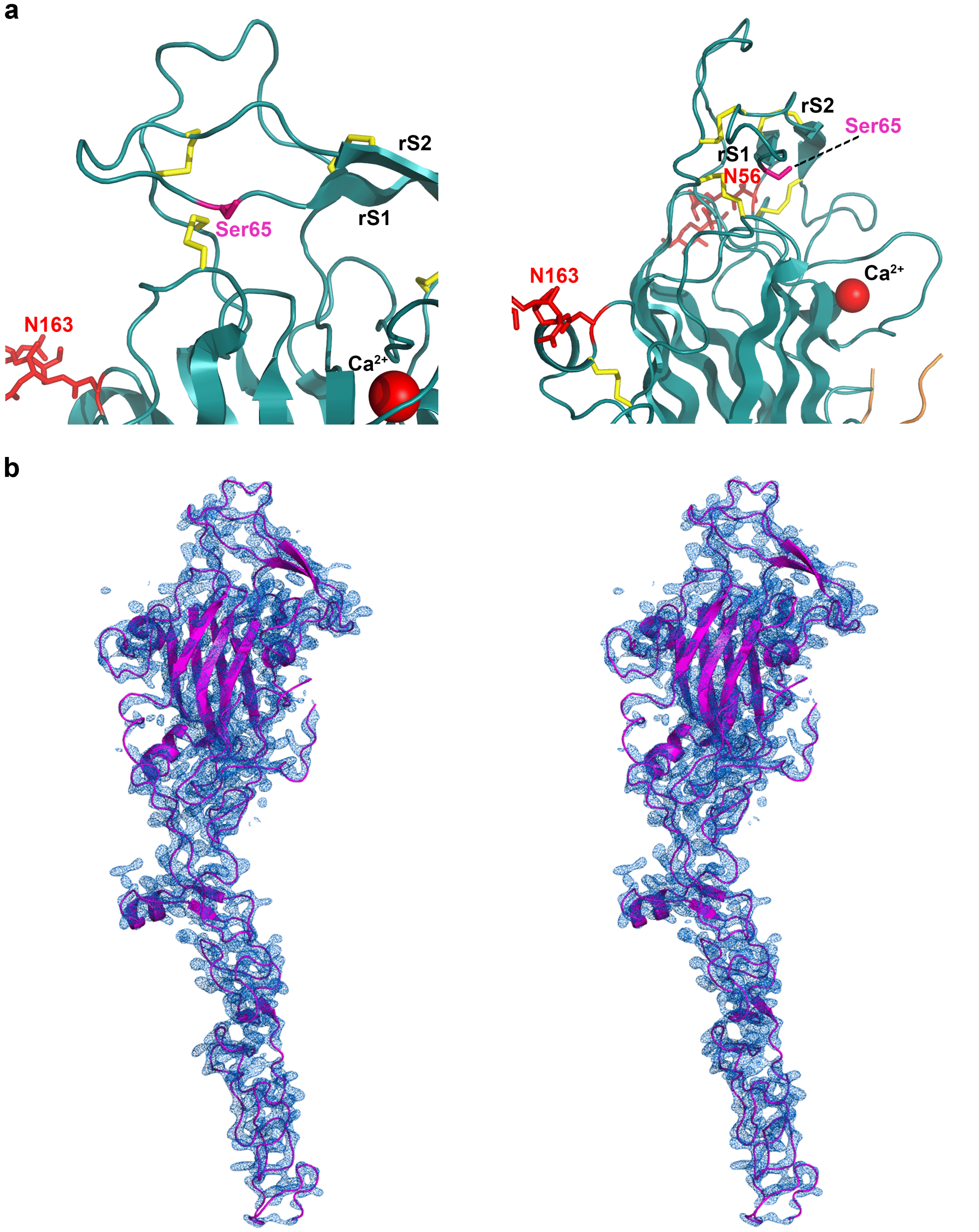

Supplementary Figure 2. Structural features of Net4-ΔC

(**a**) The conserved Ser65 is held in place by 2 unique disulfide bridges. The loop between rS1 and rS2 in the LN domain of Net4 is held in place by disulfide bridges 72-236 and 191-234, which are absent in all netrin and laminin structures published to date. In Lmβ1, the replacement of this conserved serine by arginine allows the formation of a binary complex between β1 and γ1 laminins; however, the ternary complex formation with the α1 laminin is abolished.

(**b**) Stereo image of electron density of Net4-ΔC. Net4-ΔC is represented in cartoon mode with secondary structure elements highlighted and sidechains omitted for simplicity. The 2FoFc map is contoured at 2.0σ.

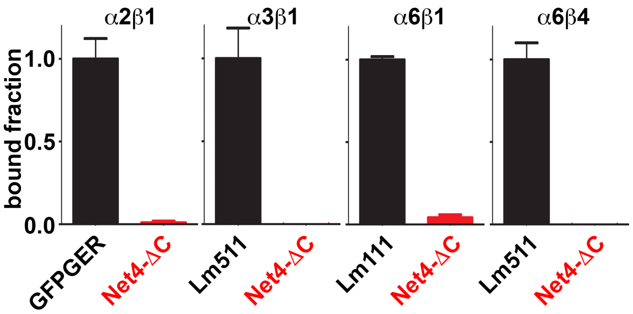

Supplementary Figure 3. Net4 binding to integrins

Solid-phase binding studies of Net4-ΔC (analyte) binding to immobilized integrin α2β1, α3β1, α6β1, and α6β4. The GFPGER peptide, laminin 511 (Lm511), and laminin 111 (LM111) were used as positive controls.
Error bars, s.d. (*n* = 3 independent technical replicates.

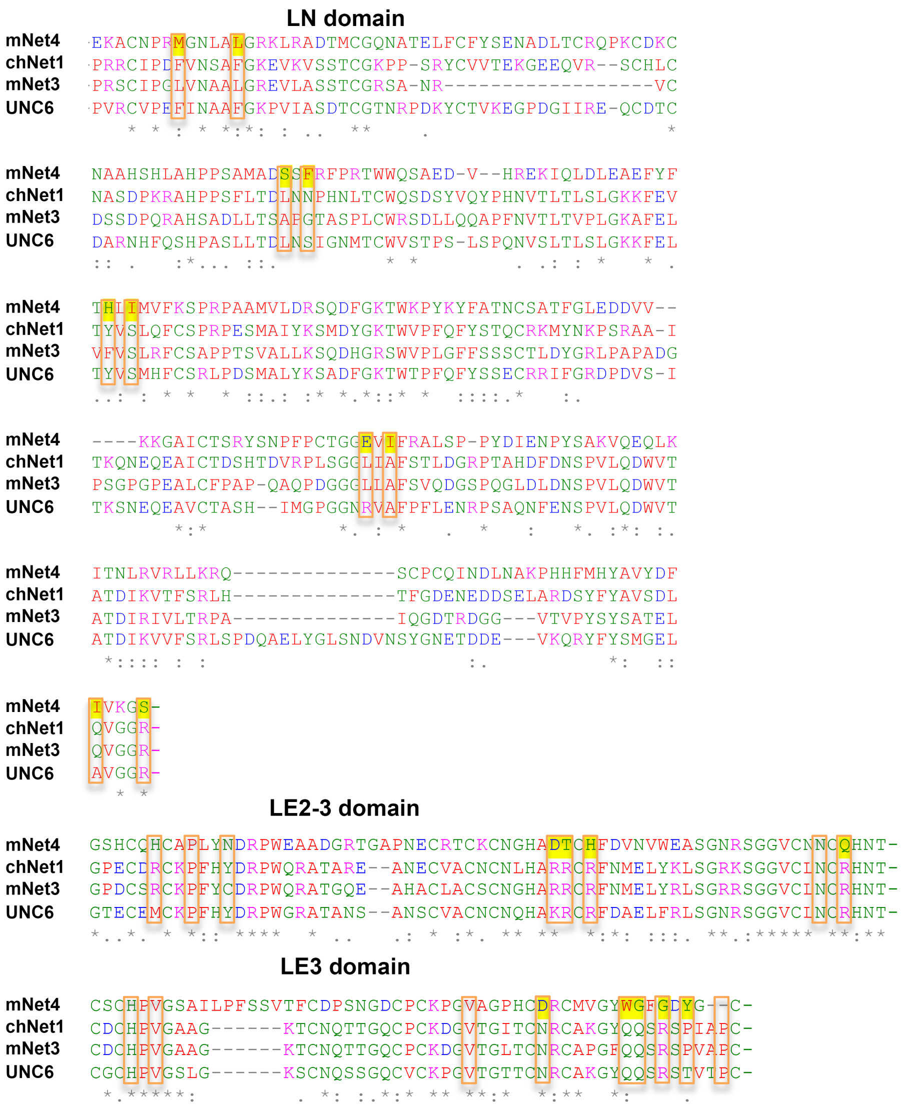

Supplementary Figure 4. Alignment of netrins and laminins

Sequence comparison between Net4 (mNet4, NP_067295), Net1 (chNet1, NP_990750), netrin-3 (mNet3, NP_035077), and unc-6 (UNC6, NP_509165). Epitopes, which are involved in DCC/neogenin and UNC5 binding, are encircled. The non-conserved amino acids within the mNet4 sequence are highlighted in yellow.

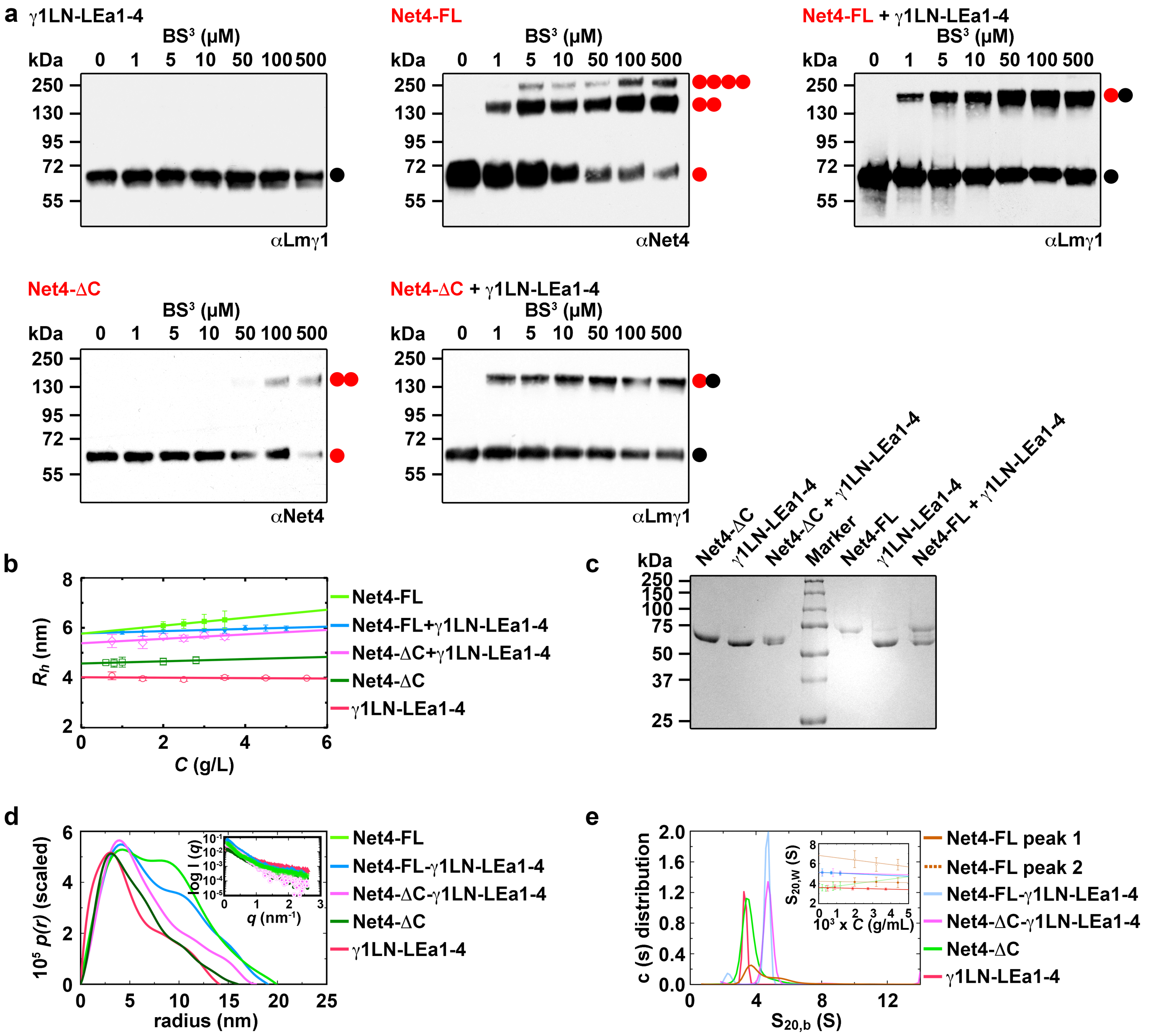

Supplementary Figure 5. Laminin γ1 interferes with the Net4-FL dimer resulting in a 1:1 complex

(**a**) Cross-linking studies of γ1LN-LEa1-4, Net4-FL, and Net4-FL in complex with γ1LN-LEa1-4. Cross-linking studies were performed using BS^3 with increasing concentrations from 0 to 500 μM and detected by either specific Net4 or Lmγ1 antibodies. The western blot represents the presence of monomeric γ1LN-LEa1-4 at all cross-linker concentrations (top left). Net4-FL reveals dimers and tetramers upon increase in BS^3 concentration (top middle). Western blot suggests that upon addition of γ1LN-LEa1-4, Net4-FL forms a 1:1 complex with γ1LN-LEa1-4 over the whole concentration spectra (top right). Data for Net4-ΔC suggests the presence of dimers at high cross-linker concentrations, whereas Net4-ΔC together with γ1LN-LEa1-4 reveals a stable heterodimeric complex.

(**b**) Dynamic light scattering analysis for Net4-ΔC, Net4-FL, γ1LN-LEa1-4 and their complexes. Data for each species were collected at concentrations ranging from 0.75 to 5 mg ml^{-1} and the hydrodynamic radius from each concentration (*y*-axis) was plotted against concentration (*x*-axis). Error bars, s.d.for each concentration (n = 3 independent technical replicates).

(**c**) SDS-PAGE analysis for SEC purified Net4-ΔC (lane 1), γ1LN-LEa1-4 (lane 2 and 6), Net4-FL (lane 5) and a complex of Net4-ΔC-γ1LN-LEa1-4 purified from SEC (lane 3) as well as the complex of Net4-FL-γ1LN-LEa1-4 (lane 7) revealed the formation of stable complexes.

(**d**) The *p*(r) distribution function analysis for Net4-ΔC, Net4-FL, γ1LN-LEa1-4 and a complex of Net4-ΔC-γ1LN-LEa1-4 as well as Net4-FL-γ1LN-LEa1-4 with raw data (*inset*) indicating maximum particle dimension on X-axis.

(**e**) Sedimentation coefficient distribution at single concentration for Net4-ΔC (1.2 mg ml^{-1}), γ1LN-LEa1-4 (1.9 mg ml^{-1}), Net4-FL (2.0 mg m^{-1}), Net4-ΔC-γ1LN-LEa1-4 (1.2 mg ml^{-1}) and Net4-FL-γ1LN-LEa1-4 (1.2 mg ml^{-1}) complex indicating that, with the exception of Net4-FL, all species are highly monodisperse. In case of Net4-FL two species are clearly visible corresponding to monomer and dimer peak. The concentration dependence of the sedimentation coefficient for each species with standard deviation is present Supplementary Table 1.

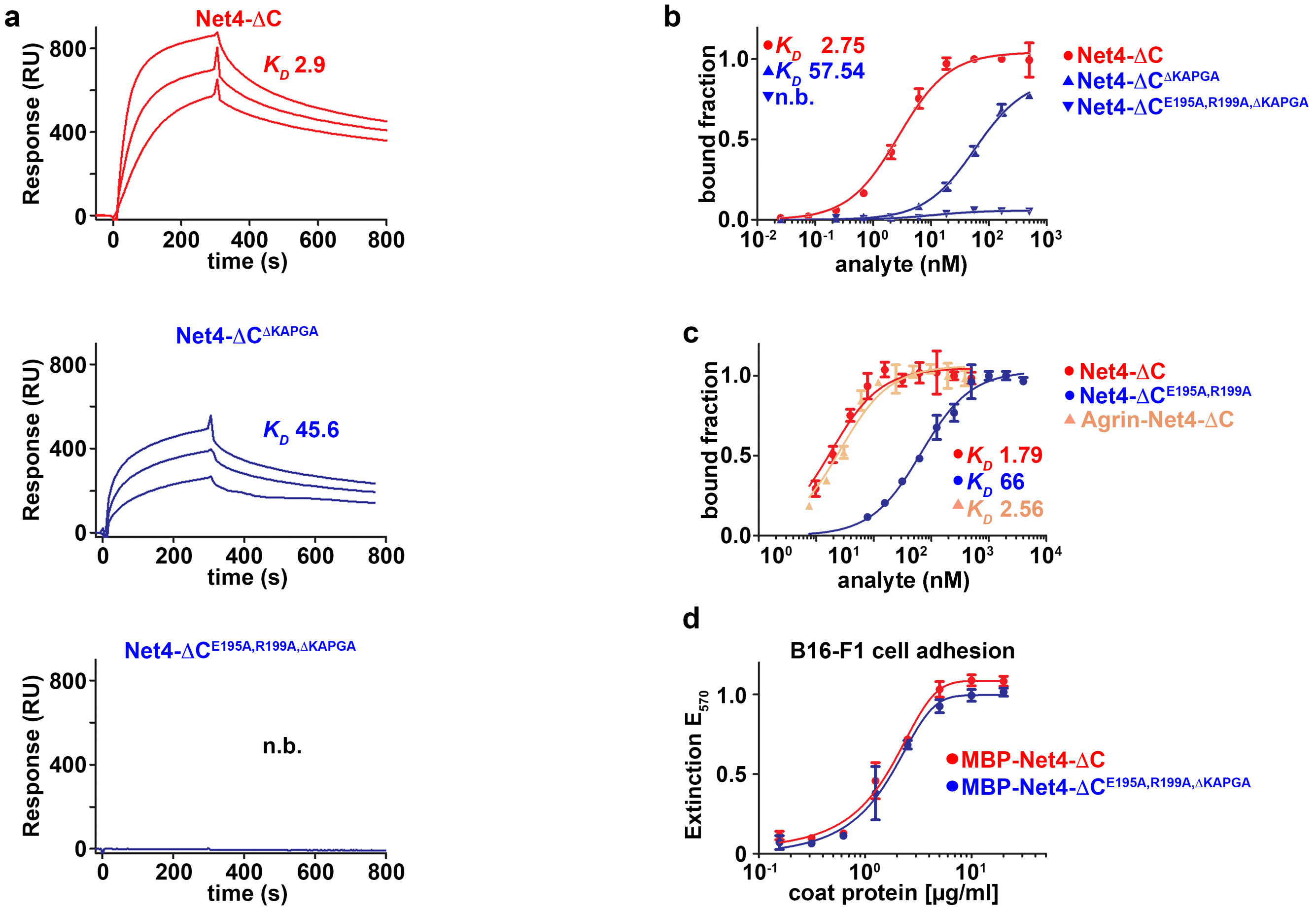

Supplementary Figure 6. Identification of the Lmγ1 binding epitopes within Net4
(**a**) SPR binding studies of Net4-ΔC, Net4-$\Delta C^{\Delta KAPGA}$, and Net4-$\Delta C^{E195A,R199A,\Delta KAPGA}$ (concentrations: 62.5, 31.25, and 15.625 nM) to immobilized γ1LN-LEa1-4.
(**b**) Solid-phase binding studies of Net4-ΔC, Net4-$\Delta C^{\Delta KAPGA}$, and Net4-$\Delta C^{E195A,R199A,\Delta KAPGA}$ (analyte) binding to immobilized γ1LN-LEa1-4.
(**c**) Solid-phase binding studies of Net4-ΔC, Net4-$\Delta C^{E195A,R199A}$, and Agrin-Net4-ΔC (analyte) binding to immobilized γ1LN-LEa1-4.
(**d**) Cell adhesion assay of mouse melanoma B16-F1 cells to immobilized MBP-Net4-ΔC and MBP-Net4-$\Delta C^{E195A,R199A,\Delta KAPGA}$.
K_D values (**a**-**c**) are shown in the graph (n.b., no binding). Error bars, s.d. (n = 3 independent technical replicates).

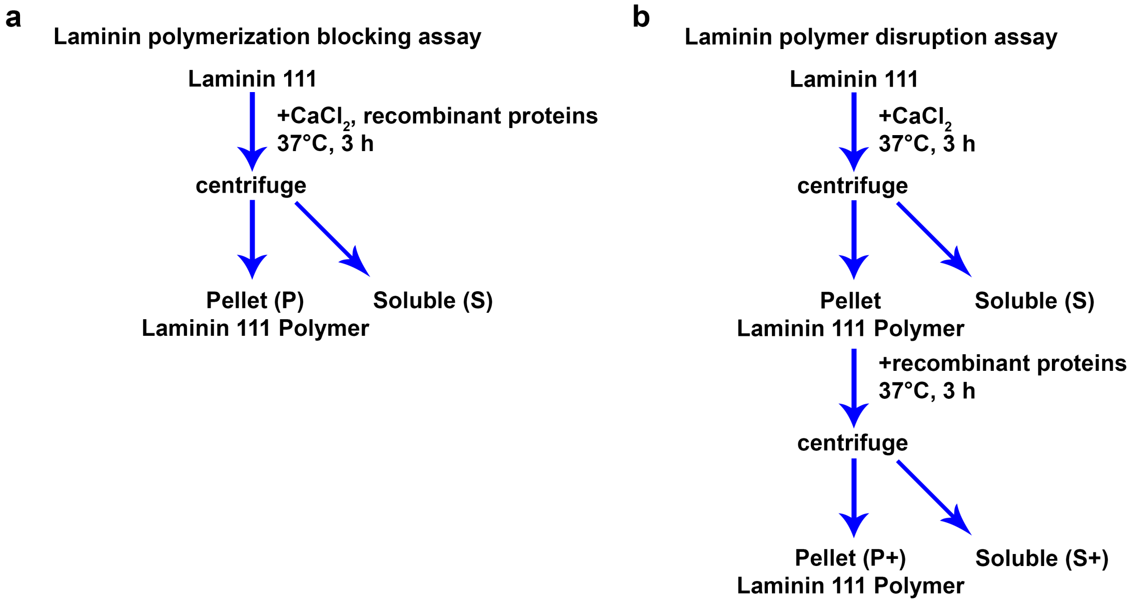

Supplementary Figure 7. Overview of the experimental procedure on laminin networks
(**a**) Procedure of the laminin polymerization assay.
(**b**) Procedure of the laminin disruption assay.

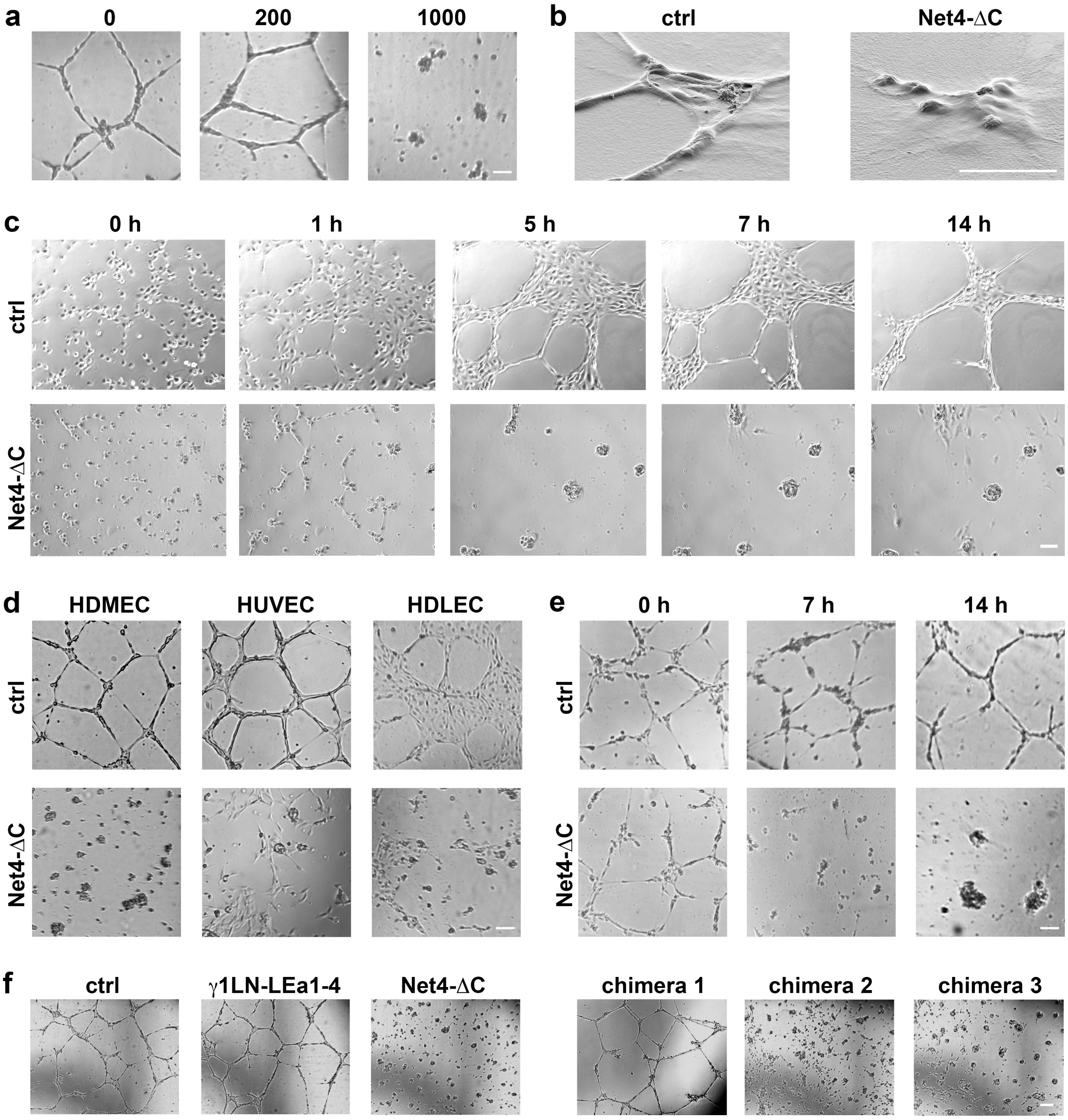

Supplementary Figure 8. The laminin network within Matrigel is necessary to establish and maintain endothelial tube-like structures
(**a**) Treatment of HDMEC cells forming tube-like structures on Matrigel with different concentrations of Net4-ΔC (0-1000 nM).
(**b**) Scanning electron microscopy images of untreated and Net4-ΔC (1 µM) treated HDMEC cells on Matrigel.
(**c**) Tube formation images of untreated and Net-ΔC-treated (1 µM) HDMEC cells at different time points (0, 1, 5, 7, and 14 h).
(**d**) Human dermal microvascular endothelial cells (HDMECs), human umbilical vein endothelial cells (HUVECs), and human dermal lymphatic endothelial cells (HDLECs) were treated with Net-ΔC (1 µM) on Matrigel.
(**e**) Preformed HDMEC tube-like structures (7 h after seeding) were treated with and without Net-ΔC (1 µM) and images were taken from 0, 7, and 14 h post treatment.
(**f**) Determination of the Net4 domains mediating the inhibition of HDMEC formed tube-like structures on Matrigel. Net4-ΔC completely inhibits the formation of tubes as well as Net4 domain swap mutants containing the laminin γ1 binding region LN–LE1 indicated by the inhibiting activity of the swap mutants Net4LN-LE2 - γ1LEa3-4 and Net4LN-LE1 - γ1LEa2-4. Treatment of endothelial cells with the laminin fragment γ1LN-LEa1-4 or the swap mutant γ1LN-LEa1 - Net4LE2-31/2 revealed no difference compared to untreated HDMECs.
Scale bars, (**a** and **c**-**f**) 100 µm, (**b**) 50 µm.

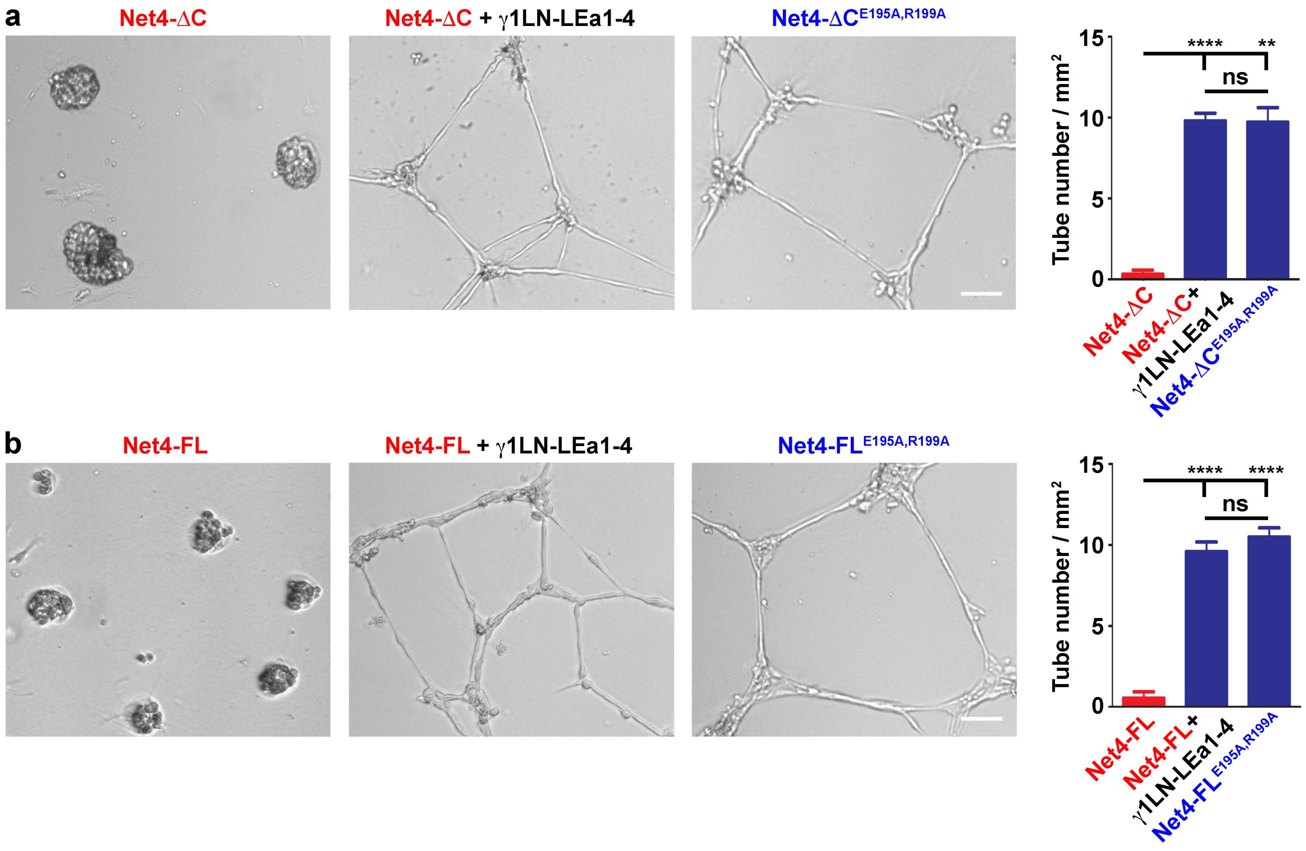

Supplementary Figure 9. Inhibition of endothelial tube-like structures through Net4
The concentration of all used proteins is 1 μM.
(**a**) HDMECs were treated with Net4-ΔC, Net4-ΔC in combination with γ1LN-LEa1-4, and Net4-ΔC^{E195A,R199A}.
Statistical analysis of the tube number per mm^2 (mean ± s.d.; n = 3; **P = 0.0015, ****P = 0.00006).
(**b**) HDMECs were treated with Net4-FL, Net4-FL in combination with γ1LN-LEa1-4, and Net4-FL^{E195A,R199A}.
Statistical analysis of the tube number per mm^2 (mean ± s.d.; n = 3; ****P = 0.00008 (Net4-FL vs. Net4-FL + γ1LN-LEa1-4), ****P = 0.00004 (Net4-FL vs. Net4-FL^{E195A,R199A})).
Scale bar, (**a** and **b**) 100 μm. Error bars, s.d. (n = 3 independent cell cultures).

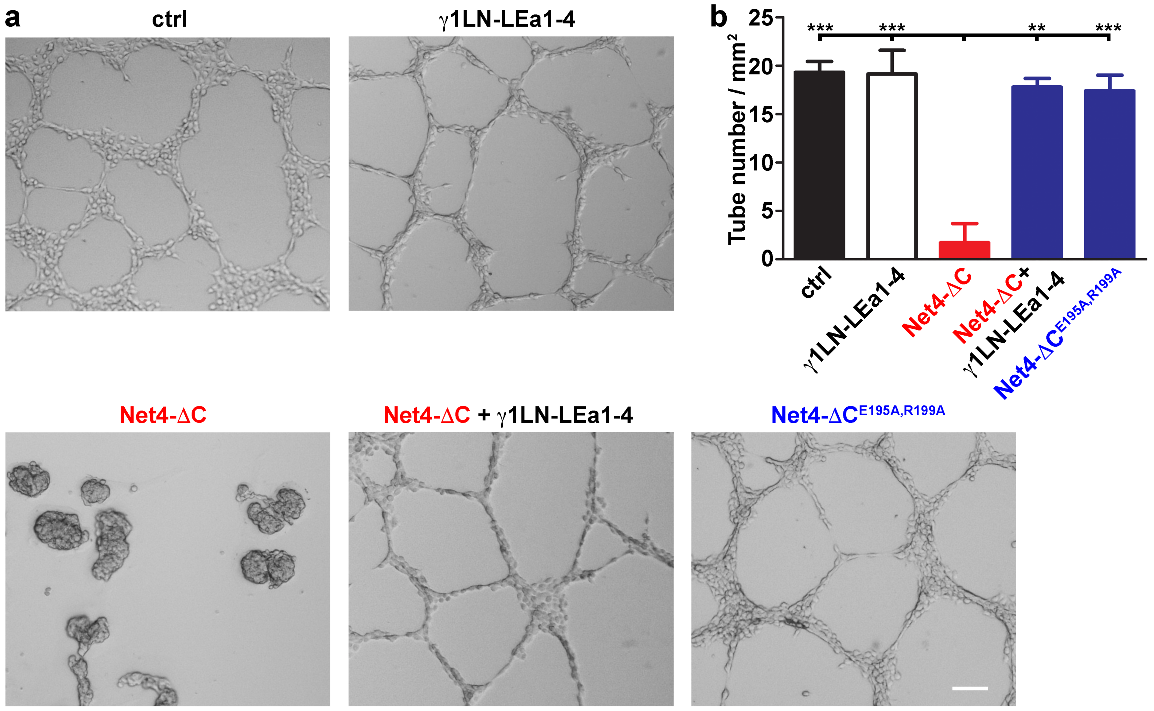

Supplementary Figure 10. Inhibition of B16-F1 tube-like structure formation through Net4
(**a**) B16-F1 cells seeded on Matrigel were treated with 1 μM γ1LN-LEa1-4, Net4-ΔC, Net4-ΔC in combination with γ1LN-LEa1-4, and the laminin-binding mutant Net4-ΔC^{E195A,R199A}.
(**b**) Statistical analysis of tube number per mm^2 (mean ± s.d.; *n* = 3; ****P* = 0.0008 (ctrl vs. Net4-ΔC), ****P* = 0.0008 (γ1LN-LEa1-4 vs. Net4-ΔC), ***P* = 0.0014 (γ1LN-LEa1-4 + Net4-ΔC vs. Net4-ΔC), ****P* = 0.0005 (Net4-ΔC^{E195A,R199A} vs. Net4-ΔC)).
Scale bar, (**a**) 100 μm. Error bars, s.d. (*n* = 3 independent cell cultures).

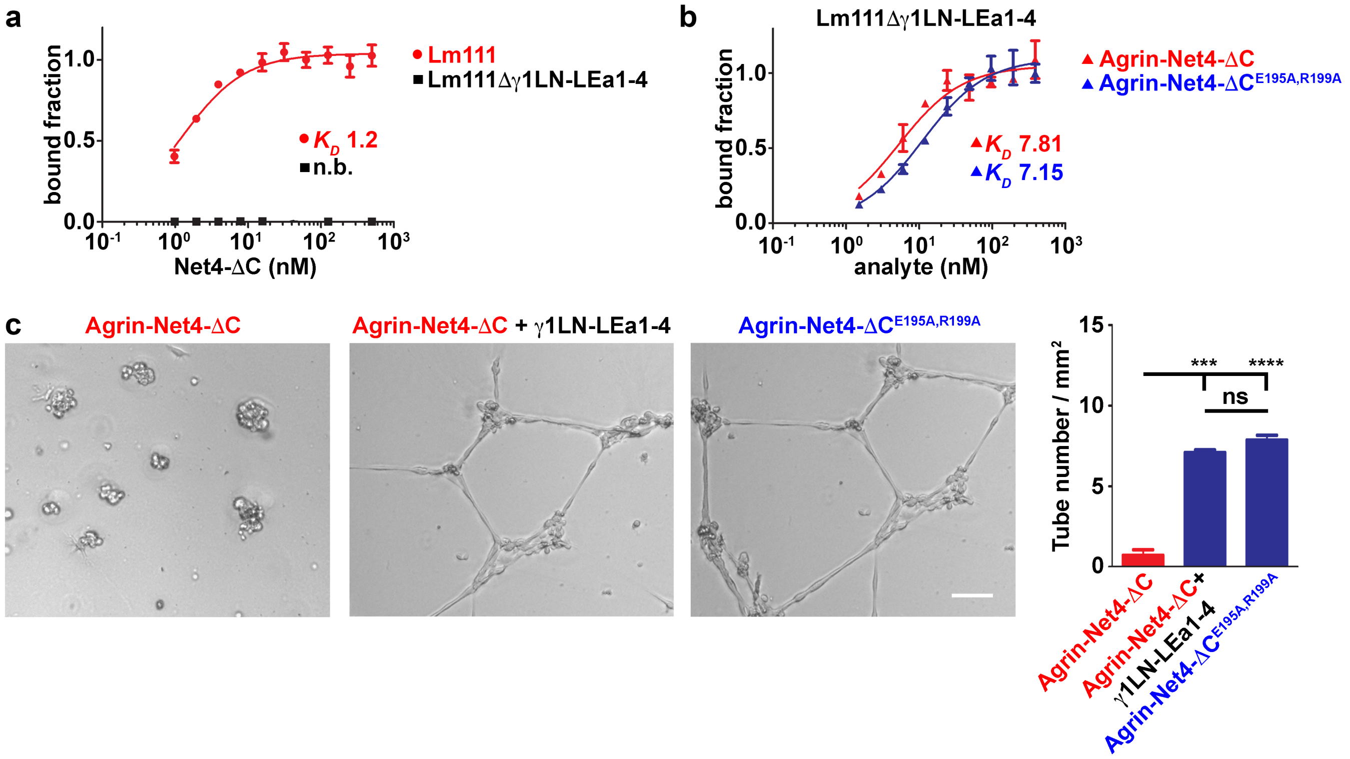

Supplementary Figure 11. Inhibitory activity of Net4 is independent from matrix deposition

(**a**) Solid-phase binding studies of Net4-ΔC, Net4-ΔC^{E195A,R199A}, and Agrin-Net4-ΔC (analyte) binding to immobilized γ1LN-LEa1-4.
(**b**) Solid-phase binding studies of Net4-ΔC (analyte) binding to immobilized laminin 111 (Lm111) and Lm111 truncated of the N-terminal γ1LN-LEa1-4 domains (Lm111Δγ1LN-LEa1-4).
K_D values are shown in the graph (n.b., no binding). Error bars (**a** and **b**), s.d. (*n* = 3 independent technical replicates).
(**c**) HDMECs were treated with 1 μM Agrin-Net4-ΔC, Agrin-Net4-ΔC in combination with γ1LN-LEa1-4, and Agrin-Net4-ΔC^{E195A,R199A}. Statistical analysis of the tube number per mm^2 (mean ± s.d.; *n* = 3; ***P = 0.00013, ****P = 0.000013). Scale bar, 100 μm. Error bars, s.d. (*n* = 3 independent cell cultures).

Name	Domains	Structure
Net4-ΔC	LN-LE1-$3^1/_2$	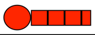
Net4-FL	LN-LE1-$3^1/_2$-NTR	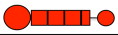
Net1-ΔC	LN-LE1-3	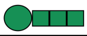
α1	α1LN-LEa1-4	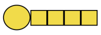
β1	β1LN-LEa1-4	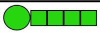
γ1	γ1LN-LEa1-4	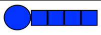
chimera 1	γ1LN-LEa1 - Net4 LE1-$3^1/_2$	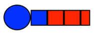
chimera 2	Net4LN-LE1-2 - γ1LEa3-4	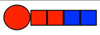
chimera 3	Net4LN-LE1 - γ1LEa2-4	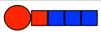
Net4-ΔC^{E195A}	LN-LE1-$3^1/_2$	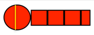
Net4-ΔC^{R199A}	LN-LE1-$3^1/_2$	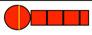
Net4-ΔC$^{\Delta KAPGA}$	LN-LE1-$3^1/_2$	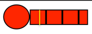
Net4-ΔC^{E195,R199}	LN-LE1-$3^1/_2$	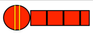
Net4-ΔC$^{E195,R199,\Delta KAPGA}$	LN-LE1-$3^1/_2$	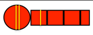
Net4-FL^{E195A,R199A}	LN-LE1-$3^1/_2$-NTR	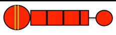

Supplementary Figure 12. Overview of the recombinant proteins used in this study
Table depicts the recombinant proteins, which have been produced for this study

Supplementary Table 1 Hydrodynamic data for Net4-ΔC, LNγ1, Net4-ΔC-γ1LN-LEa1-4, and Net4-FL-γ1LN-LEa1-4

Parameters	Net4-ΔC	Net4-FL	γ1LN-LEa1-4		Net4-ΔC-γ1LN-LEa1-4		Net4-FL-γ1LN-LEa1-4	
	Exp.[d]	Exp.	Exp.	CORAL[e]	Exp.	SASREF[e]	Exp.	SASREF[e]
r_H (nm)[a]	4.60±0.20	5.76 ± 0.02	4.02 ± 0.05	3.95 ± 0.08	5.40 ± 0.10	5.37 ± 0.02	5.77 ± 0.02	5.86 ± 0.02
r_G (nm)[b]	4.42±0.20	6.03±0.06	4.20 ± 0.04	4.12 ± 0.01	5.31 ± 0.07	5.31 ± 0.05	6.07 ± 0.04	6.00 ± 0.04
D_{max} (nm)[b]	16.00	20.0	14.1	14.2 ± 0.27	17.8	20.4 ± 0.28	19.0	21.5 ± 0.25
$S^0_{20,w}$ (S)[c]	3.51 ± 0.07	4.23 ± 0.06[f] 6.82 ± 0.41[g]	3.62 ± 0.04	3.71 ± 0.10	5.11 ± 0.04	5.32 ± 0.02	5.15 ± 0.01	5.55 ± 0.10
X[h]	-	-	-	1.08	-	1.14	-	1.45
NSD[i]	-	-	-	0.91 ± 0.12	-	0.27 ± 0.03	-	0.35 ± 0.05

[a]Experimentally determined from DLS data, with error obtained from linear regression analysis

[b]Experimentally determined from *p*(*r*) analysis by GNOM

[c]Experimentally determined using Analytical Ultracentrifuge

[d]Previously published data[2]

[e]Model-based parameters calculated from HYDROPRO, with errors obtained as the standard deviation from multiple models.

[f, g]Peak 1 and 2 respectively from Sedimentation coefficient distribution

[h]Goodness of fit parameter that represents agreement between SAXS raw data and data back calculated from models

[i]Normalized spatial discrepancy parameter representing agreement between multiple models

Methods

Biophysical analysis

The hydrodynamic radius of purified Net4-ΔC, Net4-FL, γ1LN-LEa1-4, and the complex of Net4-ΔC-γ1LN-LEa1-4 as well as the Net4-FL-γ1LN-LEa1-4 complex were measured using the Nano-S Dynamic Light Scattering system (Malvern Instruments Ltd, Malvern (UK)) as previously described[1,2], which employs a 633 nm laser and a fixed scattering angle (173°). Solutions were subjected to centrifugal filtration through a 0.1 μm filter (Millipore) prior to dilution to yield a series of solutions with concentrations in the range 0.6–5.5 mg ml^{-1}. For each measurement the protein solution was allowed to equilibrate for 4 min at 20°C prior to DLS measurements, after which five records of the DLS profiles were collected for data analyses. Sedimentation velocity experiments were performed on an Optima XL-I analytical ultracentrifuge (Beckman–Coulter, Palo Alto, CA) fitted with an An60-Ti rotor. Standard 12-mm double sector cells were loaded with 380 μL of Net4-ΔC, γ1LN-LEa1-4 and their complex as well as Net4-FL and its complex with γ1LN-LEa1-4 at multiple concentrations from 1.0 to 4.4 mg ml^{-1}) in 50 mM Tris, 200 mM NaCl buffer and 380 μL of buffer in the appropriate channels. Solutions were subjected to centrifugation between 30,000 to 40,000 rpm at 20°C. Data were recorded at 10 to 12 min intervals with absorbance and Rayleigh interference optics. The distributions were analyzed by the SEDFIT program[3,4] to obtain the weight-average sedimentation coefficient, which was then corrected to standard solvent conditions ($s_{20,w}$) using SEDNTERP[5] and the partial specific volume of 0.709 ml g^{-1} for Net4-ΔC, 0.716 ml g^{-1} for Net4-FL and 0.700 ml g^{-1} for γ1LN-LEa1-4 (considering 4 kDa for post-translational glycosylation), as well as 0.706 ml g^{-1} and 0.710 ml g^{-1} for complex of

Net4-ΔC-γ1LN-LEa1-4 and Net4-FL-γ1LN-LEa1-4 (considering 8 kDa of post-translational glycosylation for each species). The molecular weight for the complex was measured according equation 1 in[6].

Low-resolution shape determination

SAXS data were collected using a Rigaku 3-pinhole camera (S-MAX3000) equipped with a Rigaku MicroMax +002 microfocus sealed tube (Cu Kα radiation at 0.154 nm) and Confocal Max-Flux optics operating at 40 W as described previously[1,2,7]. SAXS data at 1.2, 1.6, 2.0 and 2.4 mg ml^{-1} solutions of Net4-ΔC-γ1LN-LEa1-4 complex in TBS buffer were collected in the range $0.0008 \leq q \leq 0.026$ nm with 3 h of exposure for each sample. SAXS data for Net4 were collected at 2.5, 3.0, 3.5 and 4.0 mg ml^{-1} in 50 mM Tris, 200 mM NaCl buffer, whereas data for γ1LN-LEa1-4 were collected at 1.5, 2.0, 2.5 and 3.0 mg ml^{-1}. SAXS data for Net4-FL-γ1LN-LEa1-4 were collected at 3.0, 4.0 and 5.0 mg ml^{-1}. SAXS data for each concentration and the buffer were reduced using Rigaku's SAXGUI data processing software prior to buffer subtraction from protein data using the program PRIMUS. In addition, data sets for all concentrations were merged with PRIMUS[8] to obtain a single data set. The merged data set was then analyzed by means of the GNOM program[9] to obtain the radius of gyration, r_G, the distance distribution function, $p(r)$, and the maximum particle dimension, D_{max}. To calculate high-resolution models for the complex of Net4-ΔC-γ1LN-LEa1-4, as a first step, multiple models of γ1LN-LEa1-4 were calculated using program CORAL[10] and homology models designed using SWISS-MODEL[11,12] based on the PDB structures 4AQT[13] and 1NPE[14]. Once the solution structure of γ1LN-LEa1-4 was obtained, high-resolution structure of Net4-ΔC and solution scattering data for the Net4-ΔC-γ1LN-LEa1-4 complex were used as input parameters to

calculate multiple models of the complex using program SASREF[15]. The models were imported into the MacPymol program for figure preparation. Validation of SAXS models was carried out performing HYDROPRO calculations as described previously[1,2,7]. To calculate high-resolution structures for Net4-FL-γ1LN-LEa1-4, we employed SAXS data for Net4-FL-γ1LN-LEa1-4, the high-resolution model for Net-4ΔC-γ1LN-LEa1-4 and homology models for the C-terminal domains based on the PDBs 1NPE[14] and 1UAP[16] as input data for program SASREF. We also analysed the low-resolution shape of Net4-ΔC-γ1LN-LEa1-4 complex using rotary shadowing microscopy in 50 mM Tris/HCl, 200 mM NaCl buffer as described previously[17].

AFM indentation measurements of the Matrigel matrix

Measurements were carried out using a NanoWizard® I AFM (JPK Instruments, Berlin, Germany) mounted on an optical microscope (Axiovert 200, Carl Zeiss MicroImaging GmbH, Göttingen, Germany) with a custom-made temperature unit for 37°C. In order to reduce the influence of ambient noise, the Axiovert was placed on an active isolation table (Micro 60, Halcyonics, Göttingen, Germany) and the whole setup was placed inside a 1 m^3 soundproof box. For indentation measurements silicon nitride cantilevers (MLCT, Microcantilever, Bruker, Mannheim) with a nominal spring constant of 0.1 N/m and integrated pyramidal tips with a nominal radius of 20 nm were used. The actual cantilever spring constant of each cantilever was determined more precisely by using the thermal noise method[18,19]. Every spring constant measurement was repeated three times and the arithmetic average was then used for data analysis.

2 ml of Matrigel™ Matrix (Corning, New York, United States) were plated on a pre-cooled petri dish and cured at room temperature. Afterwards, the dish was mounted on the temperature unit and 9 ml of PBS (pH 7.4) were added. On an area of 3 x 3

µm² 8 x 8 indentation curves consisting of 256 data points each were recorded on three different positions. Indentations were performed at a rate of 5 µm s^{-1} in order to probe elastic rather than viscoelastic behavior[20]. Subsequently, Net4-ΔC or Net4-$\Delta C^{E195A,R199A}$ was added and the procedure was repeated.

Young´s modulus was extracted from the approach force-indentation curves using a modified Hertz model for a pyramidal indenter with the help of JPK Data Processing software (Version 5.0.67, JPK Instruments AG, Berlin, Germany). In this model, the force (F), required to push into the samples is a quadratic function of the indentation depth (δ):

$$F = \frac{2 * tan\alpha * E}{\pi * (1 - \nu^2)} * \delta^2$$

E is the Young´s modulus; v as the Poisson´s ratio, which was set to 0.5[21] and α the tip half-opening angle (17.5° for our cantilever).

Based on the results obtained from three individual force maps for each setup, stiffness distributions (histograms) were calculated. To locate the maxima, a Gaussian function was fitted to the distributions using the Igor Pro software (Version 6.3.4.0).

SEC analysis of laminin polymer disruption

Individual recombinant laminin fragments of α1, β1, and γ1 (LN–LEa1-4), and MBP-Net4-ΔC were concentrated up to ~6µM in 20 mM Tris/HCl, pH 8.5; 150 mM NaCl. The ternary laminin complex was made by mixing equimolar quantities of the laminins for a final concentration of 2 µM and incubating at room temperature for 1 h with 2 mM $CaCl_2$. For the final run, MBP-Net4-ΔC was added in 6-fold molar excess for a laminin concentration of 1.2 µM and a MBP-Net4-ΔC concentration of 7.2 µM. All proteins and complexes were separated on a GE Healthcare superdex 200

10/300GL column, and absorbance was monitored at 280 nm. The data was scaled according to the laminin 111 complex concentrations.

Raster electron microscopy

For electron microscopy, plastic foils were put in the wells of Labtek chamber slides, coated with Matrigel™, and incubated at 37°C for 1 h. Thereafter, 20,000 cells per well were seeded and incubated for 4.5 h until the tubular network was formed. Then Net4-ΔC was added at a concentration of 1 µM for 1 h, followed by washing with PBS and fixation with 2% glutaraldehyde/2% paraformaldehyde at room temperature for 1 h. Culture samples from groups of three of one biological replicate were analyzed.

Spheroid sprouting assay

About 1,000 HUVEC cells were seeded in 20% methylcellulose (Sigma) and 80% growth medium (Cell Systems) into a 96-well plate to induce spheroid formation. After 24 h at 37°C the spheroids were harvested, centrifuged at 5000 g for 3 minutes and each 50 spheroids were covered with 250 µl 20% methylcellulose and 80% growth medium, mixed with 250 µl non-polymerized collagen gel (2 mg ml^{-1}) and seeded into a well of a 24-well plate. After polymerization of the collagen gels, Net4-ΔC (1 µM) was added. VEGF-A (20 ng ml^{-1}, 94900 Biomol) addition served as positive control. After 24 and 48 h incubation at 37°C, 10 randomly chosen spheroids were analyzed for each condition by applying bright-field microscopy (Nikon Eclipse TE2000-U). All experiments were performed in groups of three independent cultures and repeated at least twice.

Protein stoichiometry determination via cross-linking

Experiments were performed at a protein concentration of 500 nM in a final volume of 50 µL of PBS, pH 7.4. The cross-linker bis[sulfosuccinimidyl]suberate (BS^3, Pierce)

was used at concentrations from 0 to 500 µM. The reactions were allowed to incubate on ice for 1 h and were stopped by the addition of 10 µL of 1 M Tris-HCl, pH 8.0. The single cross-linking samples were afterwards analyzed via SDS-PAGE using NuPAGE® Novex® 4-12% Bis-Tris pre-cast gels followed by western blot analysis. Proteins were detected using specific antibodies against Net4 (1:1000; KR1) or a streptavidin horseradish peroxidase (HRP)-conjugate (2-1502-001, IBA) 1:100,000 for γ1LN-LEa1-4. Validation information for the Net4 antibody is presented in the work of Schneiders et., al.[22].

Solid phase binding assays

γ1LN-LEa1-4 without tag, recombinant mouse laminin 111 as well as mouse laminin 111, in which the LN-LEa1-4 domains at the laminin γ1 have been truncated (kindly provided by Peter Yurchenco), and α2β1, α3β1, α6β1 as well as α6β4 (R&D) were coated at 10 µg ml^{-1} (500 ng/well) at 4°C onto 96-well plates (NuncMaxisorb) overnight. After washing with TBS, plates were blocked with TBS containing 3% bovine serum albumin at room temperature for 2 h. Ligands were diluted to concentrations from 0.03 nM to 500 nM and incubated at room temperature for 2 h. After extensive washing with TBS, bound ligands were detected with a streptavidin horseradish peroxidase (HRP)-conjugate (2-1502-001, IBA) 1:5000. HRP was detected by Pierce TMB ELISA Substrate (Thermo Scientific™). Absorption was measured at 450 nm after stopping the reaction with 2 M sulfuric acid. A blank value corresponding to BSA coated wells was automatically subtracted.

Surface plasmon resonance binding assays

Assays were performed using a Biacore 2000 (BIAcore AB). γ1LN-LEa1-4 was coupled to a Ni-NTA chip via a fused octa-histidine (His_8)[23]. The experiments were

carried out using serial dilutions of the putative binding partner. The analyte was passed over the sensor chip with a constant flow rate of 30 ml min^{-1} for 300 s, and dissociation was measured over 500 s. Fittings of the data, overlay plots, and calculation of K_D values were done with BIAevaluation software 3.2 according to the Langmuir model for 1:1 binding.

Cell adhesion assay

Recombinant proteins (MBP-Net4-ΔC, MBP-Net4-$\Delta C^{E195A,R199A,\Delta KAPGA}$) were coated with a serial dilution (0-20 μg ml^{-1}) onto a 96-well plates at 4°C overnight. Trypsinated B16-F1 cells were resuspended in serum-free DMEM/F-12 (supplemented with 2 mM $MgCl_2$ and 1 mM $MnCl_2$) and 5 x 10^4 cells/well were seeded in triplicates. Cells were allowed to adhere to the various substrates at 37°C for 30 min. After carefully washing of non-adherent cells once with 1 x PBS, adherent cells were fixed with 1% glutaraldehyde for 15 min at room temperature before staining for 25 min with 0.1% crystal violet. Adherent cells were quantified by releasing the dye from the cells with 0.2% Triton X-100. The absorbance was measured in a spectrophotometer (Tecan) at 570 nm. A blank value corresponding to BSA coated wells was subtracted.

References

1. Patel, T.R. et al. Nano-structure of the laminin gamma-1 short arm reveals an extended and curved multidomain assembly. *Matrix Biol* **29**, 565-72 (2010).
2. Patel, T.R. et al. Determination of a molecular shape for netrin-4 from hydrodynamic and small angle X-ray scattering measurements. *Matrix Biol* **31**, 135-40 (2012).
3. Dam, J. & Schuck, P. Calculating sedimentation coefficient distributions by direct modeling of sedimentation velocity concentration profiles. *Numerical Computer Methods, Pt E* **384**, 185-212 (2004).
4. Schuck, P. Sedimentation analysis of noninteracting and self-associating solutes using numerical solutions to the Lamm equation. *Biophysical Journal* **75**, 1503-1512 (1998).
5. Laue, T.M., Shah, B.D., Ridgeway, T.M. & Pelletier, S.L. Computer-aided interpretation of analytical sedimentation data for proteins. in *Analytical Ultracentrifugation in Biochemistry and Polymer Science* (eds. Harding, S.E., Rowe, A.J. & Horton, J.C.) 90-125 (Royal Society of Chemistry, Cambridge, United Kingdom, 1992).
6. Deo, S. et al. Activation of 2' 5'-oligoadenylate synthetase by stem loops at the 5'-end of the West Nile virus genome. *PLoS One* **9**, e92545 (2014).
7. Patel, T.R. et al. Structural elucidation of full-length nidogen and the laminin-nidogen complex in solution. *Matrix Biol* **33**, 60-7 (2014).
8. Konarev, P.V., Volkov, V.V., Sokolova, A.V., Koch, M.H.J. & Svergun, D.I. PRIMUS: a Windows PC-based system for small-angle scattering data analysis. *Journal of Applied Crystallography* **36**, 1277-1282 (2003).
9. Svergun, D.I. Determination of the Regularization Parameter in Indirect-Transform Methods Using Perceptual Criteria. *Journal of Applied Crystallography* **25**, 495-503 (1992).
10. Petoukhov, M.V. et al. New developments in the program package for small-angle scattering data analysis. *J Appl Crystallogr* **45**, 342-350 (2012).
11. Arnold, K., Bordoli, L., Kopp, J. & Schwede, T. The SWISS-MODEL workspace: a web-based environment for protein structure homology modelling. *Bioinformatics* **22**, 195-201 (2006).

12. Biasini, M. et al. SWISS-MODEL: modelling protein tertiary and quaternary structure using evolutionary information. *Nucleic Acids Res* **42**, W252-8 (2014).
13. Carafoli, F., Hussain, S.A. & Hohenester, E. Crystal structures of the network-forming short-arm tips of the laminin beta1 and gamma1 chains. *PLoS One* **7**, e42473 (2012).
14. Takagi, J., Yang, Y., Liu, J.-h., Wang, J.-h. & Springer, T.A. Complex between nidogen and laminin fragments reveals a paradigmatic [beta]-propeller interface. *Nature* **424**, 969-974 (2003).
15. Petoukhov, M.V. & Svergun, D.I. Global rigid body modeling of macromolecular complexes against small-angle scattering data. *Biophys J* **89**, 1237-50 (2005).
16. Liepinsh, E. et al. NMR structure of the netrin-like domain (NTR) of human type I procollagen C-proteinase enhancer defines structural consensus of NTR domains and assesses potential proteinase inhibitory activity and ligand binding. *J Biol Chem* **278**, 25982-9 (2003).
17. Rousselle, P. et al. Laminin 5 Binds the NC-1 Domain of Type VII Collagen. *Journal of Cell Biology* **138**, 719-728 (1997).
18. Butt, H.J. & Jaschke, M. Calculation of thermal noise in atomic force microscopy. *Nanotechnology* **6**, 1-7 (1995).
19. Hutter, J.L. & Bechhoefer, J. Calibration of atomic-force microscope tips. *Review of Scientific Instruments* **64**, 1868-1873 (1993).
20. Mahaffy, R.E., Shih, C.K., MacKintosh, F.C. & Kas, J. Scanning probe-based frequency-dependent microrheology of polymer gels and biological cells. *Phys Rev Lett* **85**, 880-3 (2000).
21. Boudou, T., Ohayon, J., Picart, C. & Tracqui, P. An extended relationship for the characterization of Young's modulus and Poisson's ratio of tunable polyacrylamide gels. *Biorheology* **43**, 721-728 (2006).
22. Koch, M. et al. A novel member of the netrin family, beta-netrin, shares homology with the beta chain of laminin: identification, expression, and functional characterization. *J Cell Biol* **151**, 221-34 (2000).
23. Kimple, A.J., Muller, R.E., Siderovski, D.P. & Willard, F.S. A capture coupling method for the covalent immobilization of hexahistidine tagged proteins for surface plasmon resonance. *Methods Mol Biol* **627**, 91-100 (2010).

24. Notredame, C., Higgins, D.G. & Heringa, J. T-Coffee: A novel method for fast and accurate multiple sequence alignment. *J Mol Biol* **302**, 205-17 (2000).
