# Supplementary material for: Structural decoding of netrin-4 reveals a regulatory function towards mature basement membranes
Source: Nat Commun. 2016 Nov 30;7:13515. doi: 10.1038/ncomms13515 (PMC5141367; doi:10.1038/ncomms13515)
Supplement: Peer Review [file ncomms13515-s5.pdf]

Editorial Note: this manuscript has been previously reviewed at another journal that is not operating a transparent peer review scheme. This document only contains reviewer comments and rebuttal letters for versions considered at Nature Communications.

PEER REVIEW FILE

Reviewers' Comments:

Reviewer #1 (Remarks to the Author):

As noted in my original review of this manuscript, the authors describe the crystal structure of Netrin-4. Based on SAXS, electron microscopy and binding data, they propose a model for the Netrin-4/laminin- γ1 complex. They authors use structure-guided mutagenesis as well as an impressive variety of biochemical, biophysical, cellular and in vivo studies to show that Netrin-4, unlike Netrin-1, does not interact with DCC or Unc5 family members and instead exerts its biological effects by interacting tightly with the basement membrane component laminin-γ1. The additional data added in the revised manuscript strengthens this conclusions.

• The authors have appropriately addressed my original comments regarding the resolution of the crystallographic data.

• While it is difficult to prove a negative, as the authors noted in their response to the initial reviews, the data provided robustly supports their assertion that netrin-4 does NOT interaction with the netrin-1 receptors DCC and Unc5(A-D).

• Although the interaction between netrin-4 and laminin-γ1 had been briefly described in the previous literature, the authors have strengthened this observation and connected it to a relevant effect on the basement membrane. The comprehensive characterization of this interaction will be valuable to the field.

• The copy-editing is much improved from the original submission. The manuscript is still lengthy. I agree with the authors that this issue can be addressed post peer review in consultation with the journal.
